# Secondary Metabolites from Mariana Trench-Derived *Penicillium* sp. SCSIO41052 and Their Antimicrobial Activity

**DOI:** 10.3390/molecules31214034

**Published:** 2026-11-09

**Authors:** Qinlin Cao, Xiaoyan Pang, Yufan Zhang, Xin Sun, Xueni Wang, Junfeng Wang, Xuefeng Zhou, Bin Yang, Weijia Zhang, Yonghong Liu

**Affiliations:** 1 State Key Laboratory of Tropical Oceanography/Guangdong Key Laboratory of Marine Materia Medica, South China Sea Institute of Oceanology, Chinese Academy of Sciences, Guangzhou 510301, China; 2 University of Chinese Academy of Sciences, Beijing 100049, China; 3 Guangxi Engineering Research Center for High-Value Utilization of Guangxi-Produced Authentic Medicinal Herbs, Institute of Traditional Chinese and Zhuang-Yao Ethnic Medicine, Guangxi University of Chinese Medicine, Nanning 530200, China; 4 Institute of Deep-Sea Science and Engineering, Chinese Academy of Sciences, Sanya 572000, China

**Keywords:** linear N-acetylpeptides, natural products, *Penicillium*, antimicrobial activity

## Abstract

Compared to terrestrial environments, marine ecosystems are characterized by dynamic environmental conditions such as temperature, salinity, pressure. As major producers of marine natural products, marine-derived fungi thrive in these extreme habitats, leading to produce secondary metabolites with unprecedented chemical structures and unique bioactivities. In this study, two new linear N-acetylpeptides (**1** and **2**) and one new long-chain carboxylic acid (**3**) along with fifteen known compounds were isolated from *Penicillium* sp. SCSIO41052 separated from Mariana Trench sediment. Their structures were elucidated through spectroscopic analysis. Marfey’s analysis of the hydrolysate of **1** was performed to determine the absolute configurations of its amino acid residues. By comparing the ECD curves of **1** and **2**, it can be concluded that they have identical absolute configurations. The activity screening indicated that eight compounds (**3**, **4**, **5**, **6**, **10**, **12**, **13** and **18**) exhibited antimicrobial activity. Notably, compound **13** exhibited varying levels of antimicrobial activity against six pathogenic strains with Minimum Inhibitory Concentration (MIC) values ranging from 0.5 to 64 μg/mL, highlighting its potential antimicrobial properties. Moreover, compound **2** displayed moderate cytotoxic activity against the PC3 cell line. Compound **5** showed weak acetylcholinesterase (AchE) inhibition. These findings not only enriched the chemical structural diversity of secondary metabolites from marine fungi but also explored potential bioactive molecules, thereby laying a foundation for further research on marine medicinal resources.

## 1. Introduction

Marine ecosystems encompass some of the most extreme environments on Earth and remain largely unexplored. They harbour a vast diversity of microorganisms capable of producing bioactive compounds with enormous medicinal potential. Over the years, microbial natural products are widely recognized as an important source of drug leads [1]. A substantial proportion of clinically important drugs has originated from microbial natural products. For example, brentuximab vedotin, inspired by a marine natural product dolastatin 10, is approved for treating Hodgkin lymphoma and other CD30-positive malignancies [2].

Marine fungi are valuable and plentiful reservoirs of natural products that can be used to discover new bioactive compounds [3]. Notably, *Penicillium* fungi are recognized as a prolific source of structurally diverse secondary metabolites, exhibiting remarkable chemical diversity and a broad spectrum of biological activities. For instance, 463 structurally diverse and biologically active natural products were reported from marine-derived *Penicillium* fungi during 2021–2023 [4]. In recent years, an increasing number of novel bioactive compounds produced by marine-derived *Penicillium* species has been reported. For example, penipyranicins A–C, three novel aromatic polyketides featuring rare 4-methyl-4*H*-pyran moieties, were obtained from *Penicillium* sp. RO-11 isolated from hydrothermal spring sediments and exhibited antimicrobial activity [5]. Scabrosteroids A–D, four unprecedented steroidal heterodimers incorporating the first naturally occurring pyrrolidinone–ergosterol hybrid units, exhibited significant inhibition of NO production and moderate in vitro immunosuppressive activity [6]. In addition, a novel andrastin-type meroterpenoid, dfpenicimeroterpenoid A, featuring an unprecedented fused 6/6/6/4/5 pentacyclic skeleton was obtained from marine-derived fungus *Penicillium* sp. DF71 [7]. Collectively, these findings highlight the great potential of marine-derived *Penicillium* fungi as a source of novel bioactive natural products.

Mariana Trench, located in the Pacific Ocean, is the deepest marine environment on Earth and sustains a distinctive ecosystem characterized by microbial assemblages. Nevertheless, Mariana Trench-derived fungi remain poorly investigated, and their chemical and biological diversity is being discovered. For example, two unusual 13-membered macrocyclolipopeptides, cladosporiums A and B, and four new steroids, cladosporisteroids D−G, were obtained from the Mariana Trench-derived fungus *Cladosporium* sp. RCDB004 [8]. Four new citrinin derivatives were isolated from the Mariana Trench-derived fungus *Penicillium* sp. SCSIO41326. Among these, monankarin H exhibited weak AChE inhibitory activity [9]. In our previous study, seven new compounds including triterpene glycoside, sesquiterpene glycoside, α-pyrone derivatives and aliphatic hydrocarbon, were obtained from the Mariana Trench-derived fungus *Nigrocephalum* sp. SCSIO41049. Among these, nigrocephaloside A displayed the potent inhibitory effect on *Fusarium oxysporum* HNM1003 with MIC value of 4 μg/mL. Nigrocephalosides A and B displayed significant neuraminidase inhibitory activity with IC_50_ values of 43.6 and 33.0 μM, respectively [10]. In summary, Mariana Trench-derived fungi are of significant research value owing to their adaptation to extreme deep-sea environments, the limited understanding of their chemical diversity and the largely untapped potential for unique biological activities.

**Figure 1:**
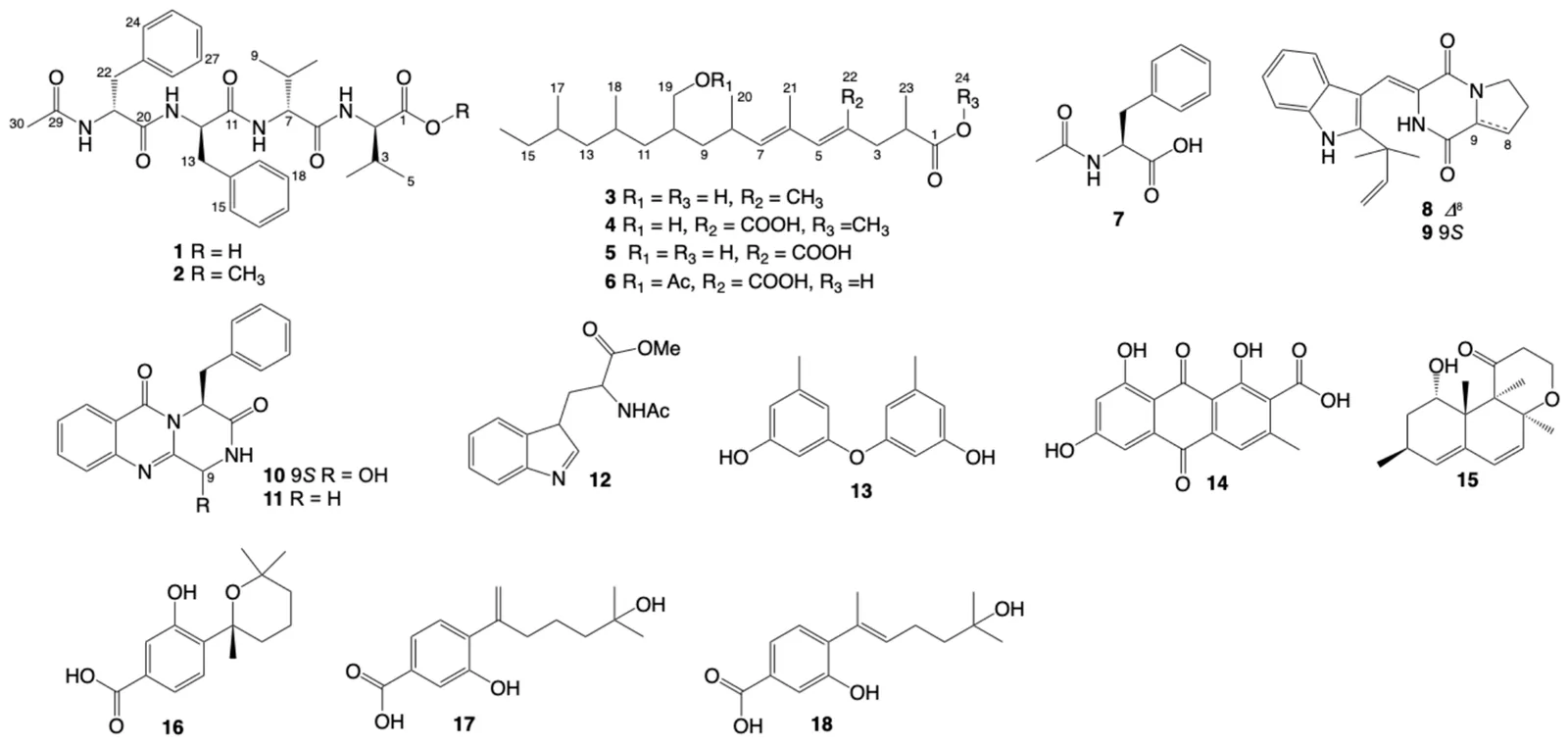
Chemical structures of compounds **1**–**18**.

Antimicrobial resistance has become a pressing global health concern, contributing substantially to the growing burden of infectious diseases, with drug-resistant infections historically estimated to cause approximately 700,000 deaths annually worldwide [11]. Among the clinical challenges associated with antimicrobial resistance, skin and soft tissue infections caused by resistant bacteria are of particular concern. Methicillin-resistant *Staphylococcus aureus* (MRSA) is an important pathogen responsible for these infections and poses considerable challenges to effective antimicrobial treatment [12]. The global spread of drug-resistant bacteria has intensified the need to discover new antibacterial agents from underexplored natural sources. In an effort to discover bioactive specialized metabolites from marine-derived *Penicillium* species, a fungus *Penicillium* sp. SCSIO41052 isolated from Mariana Trench sediment was subjected to chemical investigation. As a result, two new linear N-acetylpeptides (**1** and **2**) and one new long-chain carboxylic acid (**3**) along with fifteen known compounds (**4**–**18**) with antimicrobial, cytotoxic and AchE inhibition activities were obtained (Figure 1). Herein, we report the isolation, structural elucidation and biological evaluation of these isolated secondary metabolites.

## 2. Results and Discussion

**Figure 2:**
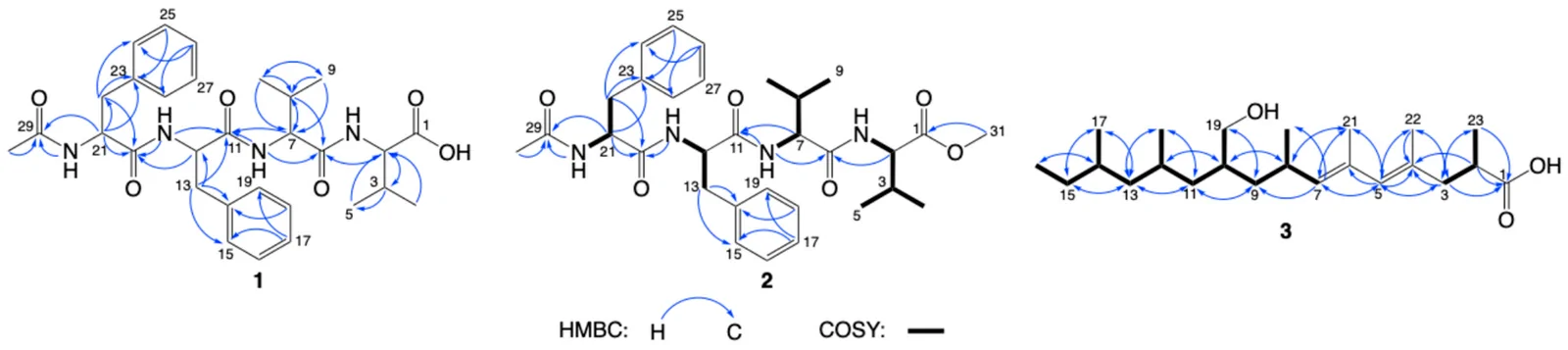
Key COSY and HMBCs of compounds **1**–**3**.

Compound **1**, a white powder, has a molecular formula of C_30_H_40_N_4_O_6_, as shown by the [M + H]^+^ positive ion high resolution electrospray ionization mass spectroscopy (HRESIMS) data at *m/z* 553.3041 (calculated for C_30_H_41_N_4_O_6_) (Figure S5), which suggests thirteen degrees of unsaturation. The ^1^H NMR spectrum of **1** (Table 1) revealed signals attributable to four *α*-protons of amino acids (*δ*_H_ 4.68, 4.41, 4.29 and 4.22), four NH groups (*δ*_H_ 8.20, 8.13, 8.03 and 7.84) and five methyl groups (*δ*_H_ 1.69, 0.72, 0.72, 0.64 and 0.62). The ^13^C NMR spectra showed signals for five carbonyl carbons (*δ*_C_ 173.0, 171.4, 170.7, 170.6, 169.1). Together, the ^1^H and ^13^C NMR spectrum indicate that **1** is a peptidic compound. Analysis of the Heteronuclear Single Quantum Coherence (HSQC) and Heteronuclear Multiple Bond Correlation (HMBC) spectra (Figure 2), which were recorded in DMSO-*d*_6_, revealed the presence of two phenylalanine fragments (Phe1 and Phe2) and two valine fragments (Val1 and Val2).

The sequence of these residues was determined through the analysis of HMBC spectra. The HMBC signals from H-2 (*δ*_H_ 4.22), NH (2) (*δ*_H_ 8.20), H-8 (*δ*_H_ 1.93) to C-6 (*δ*_C_ 170.6), from H-7 (*δ*_H_ 4.29), H_2_-13 (*δ*_H_ 2.93, 2.74), NH (2) to C-11 (*δ*_C_ 170.7) and from H-12 (*δ*_H_ 4.68), NH (3) (*δ*_H_ 7.84), H_2_-22 (*δ*_H_ 3.07, 2.83) to C-20 (*δ*_C_ 173.0) confirmed the linear sequence of **1** to be Val1-Val2-Phe1-Phe2. Moreover, the attachment of an acetyl group to NH (4) was established based on the HMBCs of H_3_-30 (*δ*_H_ 1.69), NH (4) (*δ*_H_ 8.13) and H-21 (*δ*_H_ 4.41) to C-29 (*δ*_C_ 169.1). Thus, the gross structure of **1** was figured out, as shown in Figure 1.

The absolute configurations of the amino acid residues in **1** were confirmed by Marfey’s analysis of its hydrolysates [13]. This analysis indicated that Val1, Val2, Phe1 and Phe2 were all of the D-form (Table S1). Therefore, compound **1** was determined to be phenvalpeptin A.

Compound **2** was also a white powder. The molecular formula of **2** was revealed to be C_31_H_42_N_4_O_6_ by HRESIMS (Figure S12), suggesting that **2** possessed an additional CH_2_ unit compared to **1**. With the exception of a methyl group (*δ*_H_ 3.59, *δ*_C_ 51.9), the ^1^H NMR spectrum of **2** (Table 1) is almost identical to that of **1**, which implies that **2** is an analogue of **1**. It is presumed that the carboxylic acid at C-1 underwent methyl esterification, which was confirmed by its HMBCs of H_3_-31 (*δ*_H_ 3.59) to C-1 (*δ*_C_ 171.9). The gross structure of **2** was identified using Correlation Spectroscopy (COSY), HMBC, and HSQC spectra (Figure 2), just like for **1**. By comparing the Electronic Circular Dichroism (ECD) curves of **1** (Figure S6) and **2** (Figure S13), it can be concluded that they have identical absolute configurations. Therefore, compound **2** was determined to be phenvalpeptin B.

Compound **3** was isolated as a colorless oil, and its molecular formula was established by HRESIMS to be C_23_H_42_O_3_ (Figure S19), with three degrees of unsaturation. The ^1^H NMR spectrum (Table 2) of the upfield region showed signals for seven methyl groups (*δ*_H_ 1.69, 1.66, 0.99, 0.91, 0.80, 0.80, 0.80). In the downfield region, characteristic signals were observed at *δ*_H_ 5.57 (s) and 4.96 (d, *J* = 9.5 Hz), which were associated with two trisubstituted double-bond functionalities, and at *δ*_H_ 3.62 (m, 2H), which were bonded to an oxy-methylene. In the ^13^C NMR spectrum, the characteristic signal at *δ*_C_ 177.2 was attributed to a carboxyl group. A close examination of the above ^1^H and ^13^C NMR data revealed that the structure of **3** is closely related to that of radiclonic acid (**5**) [14], except that the carboxyl group at C-4 in **5** has been replaced by a methine group (*δ*_H_ 1.69 (s, H-22) and *δ*_C_ 17.2) in **3**. This hypothesis was confirmed by the HMBCs of H_3_-22 to C-3 (*δ*_C_ 44.2), C-4 (*δ*_C_ 132.3) and C-5 (*δ*_C_ 130.5) (Figure 2). Furthermore, according to the *γ*-Gauche effect [15,16,17,18], the chemical shifts in CH_3_-21 (*δ*_C_ 21.4) and CH_3_-22 (*δ*_C_ 17.2) suggested geometries of the double bonds were assigned as 4*E* and 6*E*. Therefore, compound **3** was determined to be 4-methyl radiclonic acid.

The fifteen known compounds were identified as (3*Z*,5*E*)-8-(hydroxymethyl)-2-(3-methoxy-2-methyl-3-oxopropyl)-4,6,10,12-tetramethyltetradeca-2,4-dienoicacid (**4**) [16], radiclonic acid (**5**) [14], O-acetyl-radiclonic acid (**6**) [14], N-acetylphenylalanine (**7**) [19], brevianamide K (**8**) [20], brevianamide V (**9**) [21], brevianamide M (**10**) [22], (4*S*)-4-(2-methylethyl)-2*H*-pyrazino [2,1-b]quinazoline-3,6-(1*H*,4*H*)dione (**11**) [23], N2′-acetyltryptophan methyl ester (**12**) [24], 3,3′-dihydroxy-5,5′-dimethyldiphenyl ether (**13**) [25], endocrocin (**14**) [26], versiol (**15**) [27], sydowic acid (**16**) [28], penicibisabolane G (**17**) [29], engyodontiumone I (**18**) [30]. According to previous studies, compound **8** exhibits antibacterial activity (MIC values of 6.13 μM against *Bacillus subtilis* and *Escherichia coli*, and an MIC value of 3.06 μM against *Candida albicans*) and cytotoxicity (IC_50_ value of 16.8 μM against HGC-27 cells) [20]. Compound **5** exhibits antibacterial activity, with an MIC of 25 μg/mL against *B*. *subtilis* [15]. This finding provides a useful reference for evaluating the antimicrobial and cytotoxic activities of these compounds.

The antimicrobial effects of all compounds (**1**–**18**) were subsequently tested against five human-pathogenic bacteria (MRSA GDMCC 1.1263, *S. aureus* ATCC 29213, *Acinetobacter baumannii* BNCC254392, *Klebsiella pneumoniae* ATCC 13883 and *Enterococcus faecalis* ATCC 29212) and four plant-pathogenic fungi (*F. oxysporum* HNM1003, *F. oxysporum* f. sp. *vasinfectum*, *Alternaria solani* and *A. longipes* BNCC119020). Compounds **3**, **4**, **5**, **6**, **10**, **13** and **18** exhibited antibacterial activity against *S. aureus* with MIC values ranging from 0.5 to 128 μg/mL (Table 3). Compounds **3**, **4**, **6**, **10** and **13** also displayed significant antibacterial activity against MRSA with MIC values ranging from 0.5 to 16 μg/mL. Compound **13** was active against *A. baumannii* with an MIC value of 32 μg/mL. Compounds **4**, **5** and **13** showed moderate antifungal activity against *F. oxysporum* HNM1003 with MIC values ranging from 16 to 32 μg/mL. Compounds **4**, **6** and **13** exhibited antifungal activity against *F. oxysporum* f. sp. *vasinfectum* with MIC values ranging from 4 to 32 μg/mL. Compound **13** exhibited weak antifungal activity against *A. longipes* with MIC value of 64 μg/mL. Previous studies reported that compound **13** showed no antimicrobial activity against *B. subtilis* NBRC 3009 or *E. coli* NBRC 3301 [31], but exhibited antifungal activity against *Phytophthora nicotianae var. nicotianae* and *Sclerotinia sclerotiorum*, with IC_50_ values of 52.36 and 88.14 μg/mL [32], respectively. According to our experimental results, compound **13** demonstrated varying levels of antimicrobial activity against MRSA, *S. aureus*, *A. baumannii*, *F. oxysporum* HNM1003, *F. oxysporum* f. sp. *vasinfectum* and *A. longipes*, with MIC values ranging from 0.5 to 64 μg/mL. These findings suggest that compound **13** may exhibit selective antimicrobial activity.

Previous studies have shown that some radiclonic acid derivatives have AchE inhibitory activity [33]. Thus, compounds **3**–**6** were tested for their acetylcholinesterase inhibitory activity with tacrine as a positive control. The results revealed that compound **5** exhibited weak inhibition against AchE with IC_50_ of 103.7 μM, which was higher than the positive control (tacrine) with IC_50_ of 26.94 μM.

Moreover, we investigated the cytotoxic potential of nitrogen-containing compounds against prostate cancer cell lines. The in vitro anti-prostate cancer properties of the compounds **1**, **2**, **8**, **9**, **10**, **11** and **12** were evaluated using the Thiazolyl Blue Tetrazolium Bromide (MTT) assay on two prostate cancer cell lines (PC3 and 22RV1). The results indicated that only compound **2** at 10 μM had moderate cytotoxic activity against the PC3 cell line, resulting in 50% cell viability. As a positive control, docetaxel has been reported to exhibit antiproliferative activity against PC-3 and 22Rv1 cells, with IC_50_ values of 0.12 and 0.03 μM, respectively [34]. As not all possible biological activities have been tested, bioassays are not exhaustive. Consequently, activities other than antibacterial, AchE-inhibitory and antitumour activities may have been overlooked.

## 3. Materials and Methods

### 3.1. General Experimental Procedures

Optical rotations were recorded using an Anton Paar MPC 500 polarimeter (Waltham, MA, USA). NMR spectra were obtained using a Bruker Avance-500 MHz spectrometer (Bruker, Billerica, MA, USA), with tetramethylsilane acting as the internal standard. The HRESIMS data were obtained on a Bruker maXis Q-TOF mass spectrometer (Bruker, Billerica, MA, USA). UV and ECD spectra were collected on a Chirascan circular dichroism spectrometer (Applied Photophysics, Leatherhead, Surrey, UK). Semipreparative high-performance liquid chromatography (HPLC) was conducted on a Hitachi Primaide system equipped with a DAD detector (Hitachi Ltd., Kyoto, Japan). Separation was facilitated using an ODS column (NanoChrom-pack ODS-A/Shim-pack GIS, 10 × 250 mm, 5 μm) (Nanochrom Technologies Co. Ltd., Suzhou, China). Column chromatography (CC) was accomplished with silica gel (200–300 mesh) (Qingdao Marine Chemical Factory, Qingdao, China).

### 3.2. Fungus Strain

The fungus *Penicillium* sp. SCSIO41052 was collected from deep-sea sediment in the Mariana Trench at the depth of 10,026.2 m (11.1572° N, 141.6565° E). The ITS1-5.8S-ITS2 sequence region (563 base pairs, accession number QB034063) of strain SCSIO41052 was amplified by PCR and DNA sequencing revealed that it is closely similar to that of *Penicillium oxalicum* (accession no. NR121232.1) with 100% sequence identity and 99% query coverage. Phylogenetic analysis was conducted to further assess its relationship with related *Penicillium* species (Figure S21). Thus, the strain was identified as *Penicillium* sp. SCSIO41052. The producing strain is conserved at the Guangdong Key Laboratory of Marine Materia Medica.

### 3.3. Fermentation, Extraction and Isolation

A large-scale culture (15 kg) was grown in a rice solid medium at 26 °C for 30 days. The culture broth was extracted four times with ethyl acetate (17 L per extraction). Each extraction was performed for 2 days at 25 °C with stirring and ultrasonication, using an approximately 1:1 (*v*/*v*) ratio of culture broth to ethyl acetate. The extracts from the four extractions were combined and then concentrated under reduced pressure to obtain an ethyl acetate extract (194.0 g). The extract was mixed with silica gel (276.6 g, 100–200 mesh) for dry loading and subjected to normal-phase silica gel column chromatography using a glass column (10 cm i.d. × 40 cm bed height) with an increasing gradient of methanol (MeOH)- dichloromethane (DCM) (*v*/*v*, 1:100 to 1:1) as the mobile phase, which resulted in six fractions (Fr.1–6). Fr.1 (9.0 g) was partitioned using medium-pressure liquid chromatography with a linear gradient ranging from 0% to 100% methanol, yielding thirteen subfractions (Fr.1.1–1.13). Fr.1.5 was further separated by semi-preparative HPLC to obtain compound **14** (11.58 mg, 40:60 acetonitrile (MeCN)/H_2_O, 2.5 mL/min, *t*_R_ = 19.8 min). Fr.1.6 was further purified by semi-preparative HPLC to obtain compounds **8** (8.23 mg, 60:40 MeOH/H_2_O, containing 0.4% formic acid, 2.5 mL/min, *t*_R_ = 19.0 min), **9** (7.54 mg, 45:55 recycled MeCN/H_2_O, containing 0.6% formic acid, 2.5 mL/min, *t*_R_ = 17.5 min), **10** (6.12 mg, 55:45 MeOH/H_2_O, containing 0.4% formic acid, 2.5 mL/min, *t*_R_ = 13.0 min), **11** (7.25 mg, 45:55 recycled MeCN/H_2_O, containing 0.6% formic acid, 2.5 mL/min, *t*_R_ = 13.1 min) and **12** (6.11 mg, 56:44 MeOH/H_2_O, containing 0.4% formic acid, 2.5 mL/min, *t*_R_ = 28.5 min). Fr.1.13 was further purified by semi-preparative HPLC to obtain compound **3** (16.29 mg, 85:15 MeOH/H_2_O, containing 0.4% formic acid, 2.5 mL/min, *t*_R_ = 11.0 min). Fr.5 (4.9 g) was subjected to C18 reversed-phase (RP-18) silica gel eluted with MeOH/H_2_O (*v*/*v*, 0:100 to 100:0) to get sixteen subfractions (Fr.5.1–5.16). Compound **7** (7.42 mg) was separated from Fr.5.2 using HPLC with a 34:66 MeOH/H_2_O mixture at a flow rate of 2.5 mL/min and a retention time of 19.3 min. Fr.5.8 was separated on HPLC to collect compound **13** (8.96 mg, 43:57 recycled MeCN/H_2_O, 2.5 mL/min, *t*_R_ = 13.1 min). Fr.5.16 was further purified by semi-preparative RP HPLC. The elution was performed with 78% aqueous MeOH (containing 0.6% formic acid) at a flow rate of 2.5 mL/min to yield compounds **4** (21.21 mg, *t*_R_ = 20.3 min), **5** (206.76 mg, *t*_R_ = 13.7 min) and **6** (21.30 mg, *t*_R_ = 25.6 min). Fr.6 (2.3 g) was subjected to ODS chromatography and eluted with methanol: water (*v*/*v*, 0:100 to 100:0) to produce thirteen fractions (Fr.6.1–6.13). Fr.6.7 was obtained by RP HPLC (*v*/*v*, 28:72 recycled MeCN/H_2_O, containing 0.4% formic acid, 2.5 mL/min) to produce compound **18** (7.14 mg, *t*_R_ = 24.6 min). Similarly, Fr.6.10 was eluted with a MeOH 53% aqueous solution (containing 0.6% formic acid) to purify compounds **16** (7.90 mg, *t*_R_ = 21.7 min) and **17** (5.80 mg, *t*_R_ = 24.0 min). Fr.6.13 was further purified by semi-preparative HPLC to obtain compounds **1** (5.06 mg, 68:32 MeOH/H_2_O, containing 0.4% formic acid, 3.0 mL/min, *t*_R_ = 15.0 min), **2** (5.34 mg, 50:50 MeCN/H_2_O, containing 0.4% formic acid, 3.0 mL/min, *t*_R_ = 21.7 min) and **15** (24.77 mg, 50:50 MeCN/H_2_O, containing 0.4% formic acid, 3.0 mL/min, *t*_R_ = 30.7 min). Preparative HPLC was carried out using a NanoChrom-Pack ODS-A column (10 × 250 mm, 5 μm), with UV detection at 210 nm.

Phenvalpeptin A (**1**): white powder; [α]_25 D_ +7.0 (c 0.1, MeOH); UV (MeOH) *λ*max (log ε) 200 nm (3.57), 252 nm (2.56), 313 nm (2.32); ECD (0.54 mM, MeOH) *λ*max (Δ*ε*) 223 (–2.26) nm; ^1^H and ^13^C NMR data see Table 1; HRESIMS *m/z* 553.3041 [M + H]^+^ (calcd for C_30_H_41_N_4_O_6_, 553.3021).

Phenvalpeptin B (**2**): white powder; [α]_25 D_ +15.0 (c 0.1, MeOH); UV (MeOH) *λ*max (log ε) 200 nm (3.57), 282 nm (2.15); ECD (0.53 mM, MeOH) *λ*max (Δ*ε*) 222 (–1.71) nm; ^1^H and ^13^C NMR data see Table 1; HRESIMS *m/z* 567.3202 [M + H]^+^ (calcd for C_31_H_43_N_4_O_6_, 567.3177).

4-methyl radiclonic acid (**3**): colorless oil; [α]_25 D_ –15.4 (c 1.0, MeOH); ^1^H and ^13^C NMR data see Table 2; HRESIMS *m/z* 365.3066 [M − H]^–^ (calcd for C_23_H_41_O_3_, 365.3061).

### 3.4. Absolute Configurations Assignments by the Advanced Marfey’s Analysis

Compound **1** (0.5 mg) was hydrolyzed by treatment with 6 M HCl (500 μL) and 1,4-dioxane (100 μL) at 110 °C for 16 h. The hydrolyzed product was evaporated to dryness, after which 100 µL of 1% *w*/*v* in L-FDAA and 20 µL of 1 M NaHCO_3_ were added. The mixture was then heated to 40 °C for 1.5 h, cooled to room temperature, and neutralized with 20 µL of 1 M HCl. The products were then evaporated to dryness and analysed by RP HPLC. The same procedure described above was followed to derivatise authentic standards of L-Val, D-Val, L-Phe and D-Phe with FDAA to generate reference derivatives. Marfey’s derivatives 1 was analyzed by RP HPLC using the following chromatographic conditions: Cosmosil 5C_18_-MS-II column (*φ*10 × 250 mm, Nacalai Tesque, Kyoto, Japan); flow rate 2.0 mL/min; mobile phase A (0.3% formic acid in HPLC-grade water), mobile phase B (MeCN); gradient: 0–60.0 min, 10–60% B; 60.1–67.0 min, 10% B. The retention times (min) of the derivatives of the authentic standards are shown below: L-Val (46.2), D-Val (51.8); L-Phe (52.1); D-Phe (55.8). Meanwhile, the HPLC retention times (min) of the derivatives from the hydrolysate of 1 were as follows: D-Val (51.8); D-Phe (55.8).

### 3.5. Antimicrobial Activity Tests

Antimicrobial assays were conducted on compounds **1**–**18** against five species of human-pathogenic bacteria (MRSA, *S. aureus*, *A. baumannii*, *K. pneumoniae* and *E. faecalis*) and four species of plant-pathogenic fungi (*F. oxysporum* HNM1003, *F. oxysporum* f. sp. *vasinfectum*, *A. solani* and *A. longipes*) by the agar disc diffusion method. The MIC values of the active compounds that exhibited an apparent antimicrobial zone were evaluated on 96-well polystyrene plates using the microdilution method [35].

#### 3.5.1. Agar Disc Diffusion Assay

Antibacterial activity against human pathogenic bacteria. The bacterial strains were first activated by inoculating each strain into a 500 mL Erlenmeyer flask containing 100 mL of LB broth, followed by incubation at 25 °C with shaking for 24–48 h. The resulting bacterial suspensions were evenly spread onto LB agar plates using sterile cotton swabs. Each test compound was dissolved in DMSO to a concentration of 25 μg/μL. An aliquot of 2 μL of each compound solution (50 μg) was applied to a sterile filter paper disc (6 mm in diameter), which was then placed upside down on the agar surface. Gentamicin was used as the positive control, and DMSO served as the negative control. The plates were incubated at 25 °C for 24–48 h, after which the diameters of the inhibition zones were measured.

Antifungal activity against phytopathogenic fungi. The fungal strains were activated on MB agar plates at 25 °C for 3 days. Each test compound was dissolved in DMSO to a concentration of 25 μg/μL. An aliquot of 2 μL of each compound solution (50 μg) was applied to a sterile filter paper disc (6 mm in diameter). Four discs were placed at equidistant positions around the periphery of an MB agar plate. A square agar plug (approximately 1 cm × 1 cm) containing actively growing fungal mycelia was excised from the fungal culture using a sterile scalpel and placed upside down in the center of the plate. The distances between the fungal plug and the four discs were kept equal. Nystatin was used as the positive control, and DMSO served as the negative control. The plates were incubated at 25 °C for 2–3 days, and the antifungal activity of each compound was assessed by observing the inhibition of fungal growth.

#### 3.5.2. Determination of Minimum Inhibitory Concentrations (MICs)

The MICs of the test compounds were determined using the 96-well microdilution method. Each compound was dissolved in DMSO and subjected to serial twofold dilution in Eppendorf (EP) tubes to obtain the following concentrations: 1.28, 0.64, 0.32, 0.16, 0.08, 0.04, 0.02, 0.01, 0.005, 0.0025, and 0.00125 mg/mL. Each assay was performed in triplicate, with a final volume of 200 μL per well. Each well contained 90 μL of the microbial suspension at the appropriate working concentration, 10 μL of the test compound solution at the designated concentration, and 100 μL of autoclaved LB broth (for bacteria) or MB medium (for fungi). Gentamicin and nystatin were used as positive controls for bacteria and fungi, respectively, while DMSO served as the negative control. The plates were sealed with PE sealing film and incubated at 25 °C for 2–3 days. The absorbance at 600 nm (OD_600_) was measured using a microplate reader every 24 h. The MIC was defined as the lowest concentration of a test compound that resulted in a marked reduction in absorbance compared with the negative control.

### 3.6. Acetylcholinesterase Inhibition Assay

To determine the effects of the compounds on AChE enzyme activity, stock solutions of the compounds were prepared. In accordance with the information acquired through the studies, concentration/activity percentage graphs were drawn and IC_50_ values were calculated. We determined the AChE activity of the linear N-acetylpeptides and long-chain carboxylic acid derivatives (**1**–**6**) using the method of Ellman et al. [36].

Each compound (1 mg) was dissolved in 20 μL of DMSO. An aliquot of 2 μL of the resulting solution was transferred to an EP tube containing 98 μL of PBS and mixed thoroughly. The resulting solution was used to achieve a final sample concentration of 50 μg/mL in each well. The solutions were stored at 4 °C. Tacrine was used as the positive control and prepared in the same methods as the test compounds. A 2% DMSO solution was prepared by adding 20 μL of DMSO to 980 μL of PBS. AChE derived from fly heads was dissolved in phosphate-buffered saline (PBS, pH 8.0) to prepare an enzyme solution at a concentration of 0.1 U/mL. Acetylthiocholine iodide and 5,5′-dithiobis(2-nitrobenzoic acid) (DTNB) were separately dissolved in PBS to prepare solutions at concentrations of 6.25 mM. All solutions were stored at 4 °C. The assay was performed using the following groups: the test group (A), consisting of 10 μL of the test sample solution, 40 μL of the enzyme solution, and 110 μL of PBS; the sample control group (A_0_), consisting of 10 μL of the test sample solution and 150 μL of PBS; the negative control group (B), consisting of 10 μL of 2% DMSO, 40 μL of the enzyme solution, and 110 μL of PBS; and the blank control group (B_0_), consisting of 10 μL of 2% DMSO and 150 μL of PBS. The positive control group consisted of 10 μL of the tacrine solution, 40 μL of the enzyme solution, and 110 μL of PBS. The mixtures were added to a 96-well microplate and mixed thoroughly, followed by incubation at 30 °C for 20 min. Subsequently, 20 μL each of acetylthiocholine iodide solution and DTNB solution were added to each well, and the plate was incubated at 30 °C for an additional 30 min. The absorbance was then measured at 405 nm using a microplate reader. The AChE inhibitory rate was calculated using the following equation:

Inhibition rate (%) = [(B − B_0_) − (A − A_0_)]/(B − B_0_) × 100%

Compounds exhibiting more than 60% AChE inhibition were further evaluated to determine their IC_50_. Five final sample concentrations (50, 25, 12.5, 6.25, and 3.125 μg/mL) were tested. Tacrine was tested at the same final concentration levels as the test compounds. For compounds with lower activity, a suitable increase in the gradient should be applied. All experiments were performed in triplicate, following the procedure described above, and the AChE inhibitory rates were calculated. Concentration–response curves were plotted using GraphPad Prism 9.5.1 software with the logarithm of the compound concentration on the x-axis. The IC_50_ values were determined by regression analysis.

### 3.7. 22Rv1 and PC3 Assay

The in vitro cytotoxic potential of the nitrogen-containing compounds (**1**–**2**, **8**–**12**) was assessed using an MTT assay against two human prostate carcinoma cell lines (PC3 and 22RV1). The PC-3 and 22Rv1 prostate cancer cell lines were supplied by the Cell Bank/Stem Cell Bank of the Chinese Academy of Sciences (Shanghai, China). Docetaxel was used as the positive control. In each group, the cancer cells were first seeded in 96-well plates at a density of 4 × 10^4^ cells per well and then incubated for 24 h at 37 °C in an atmospheric CO_2_ concentration of 5%. The culture medium was then removed and replaced with 100 μL of RPMI medium containing the test compounds at a concentration of 10 μM for preliminary cytotoxicity screening. The cell lines were then incubated for 24 h in a humidified incubator at 37 °C with 5% CO_2_. Subsequently, 150 µL of MTT solution (1 mg/mL) was added to each well, replacing the media-containing compounds. Following a 4h incubation period, the MTT reagent was removed and 200 µL of DMSO was added to dissolve the formazan crystals. The resulting colour intensity was then measured at a wavelength of 540 nm using a BioTek Synergy H1 instrument (BioTek, Winooski, VT, USA). The calculation of IC_50_ was carried out using the equation of the Boltzmann sigmoidal concentration-response curve. The software used was GraphPad Prism 9.5.1 (La Jolla, CA, USA) [37].

## 4. Conclusions

In conclusion, a total of eighteen compounds were isolated from the fungus *Penicillium* sp. SCSIO41052. Among them, two new linear N-acetylpeptides and one new long-chain carboxylic acid were obtained. The structures of these isolates were identified through a combination of NMR, HRESIMS and the advanced Marfey’s analysis. Eight compounds (**3**, **4**, **5**, **6**, **10**, **12**, **13** and **18**) displayed antimicrobial activity. Remarkably, compound **13** exhibited varying levels of antimicrobial activity against MRSA, *S. aureus*, *A. baumannii*, *F. oxysporum* HNM1003, *F. oxysporum* f. sp. *vasinfectum* and *A. longipes*, with MIC values ranging from 0.5 to 64 μg/mL, highlighting its potential in antimicrobial property. Moreover, compound **5** showed weak AchE inhibition with IC_50_ of 103.7 μM. Compound **2** displayed weak cytotoxic activity against the PC3 cell line. In summary, our research enriched the diversity of linear lipopeptides and fatty acids and revealed the potential of diphenyl ether and radiclonic acid derivatives acted as antimicrobial treatments.

## Figures and Tables

**Table 1:** ^1^H (500 MHz) and ^13^C (125 MHz) NMR data of compounds **1**–**2** in DMSO-*d*_6_ (*δ* in ppm).

Amino Acid	No.	1		2	
		*δ*_C_, Type	*δ*_H_ (*J* in Hz)	*δ*_C_, Type	*δ*_H_ (*J* in Hz)
Val1	1	171.4, C		171.9, C	
	2	57.1, CH	4.22, m	57.1, CH	4.21, dd(9.0, 6.0)
	3	30.6, CH	1.82, dt(13.5, 7.0)	30.4, CH	1.83, dq(13.0, 6.5)
	4	17.3, CH_3_	0.55, d(7.0)	17.4, CH_3_	0.58, d(7.0)
	5	17.5, CH_3_	0.72, d(6.5)	17.6, CH_3_	0.64, d(6.5)
	NH (1)		8.03, d(8.5)		8.09, d(8.5)
Val2	6	170.6, C		170.8, C	
	7	57.4, CH	4.29, m	57.6, CH	4.27, dd(8.5, 5.5)
	8	30.7, CH	1.93, dt(13.0, 6.5)	30.6, CH	1.94, dq(13.5, 7.0)
	9	19.2, CH_3_	0.72, d(6.5)	19.2, CH_3_	0.73, d(7.0)
	10	19.2, CH_3_	0.64, d(6.5)	19.1, CH_3_	0.73, d(6.5)
	NH (2)		8.20, d(6.5)		7.88, d(9.5)
Phe1	11	170.7, C		170.9, C	
	12	53.8, CH	4.68, m	53.5, CH	4.49, m
	13	38.1, CH_2_	2.93, dd(14.0, 6.0) 2.74, dd(13.5, 9.5)	36.7, CH_2_	3.05, dd(13.5, 5.0) 2.87, dd(14.0, 10.0)
	14	137.8, C		137.2, C	
	15	129.2, CH	7.21, m	129.1, CH	7.20, m
	16	128.0, CH	7.24, m	128.2, CH	7.24, m
	17	126.2, CH	7.16, m	126.2, CH	7.18, m
	18	128.0, CH	7.24, m	128.2, CH	7.24, m
	19	129.2, CH	7.21, m	129.1, CH	7.20, m
	NH (3)		7.84, d(8.5)		8.43, d(8.0)
Phe2	20	173.0, C		171.6, C	
	21	53.5, CH	4.41, m	53.9, CH	4.69, dt(14.0, 5.5)
	22	37.0, CH_2_	3.07, dd(14.0, 5.0) 2.83, dd(13.5, 10.0)	38.2, CH_2_	2.93, dd(13.5, 5.5) 2.74, dd(13.5, 9.5)
	23	137.8, C		137.8, C	
	24	129.2, CH	7.21, m	129.2, CH	7.20, m
	25	128.1, CH	7.24, m	128.0, CH	7.24, m
	26	126.3, CH	7.16, m	126.5, CH	7.18, m
	27	128.1, CH	7.24, m	128.0, CH	7.24, m
	28	129.2, CH	7.21, m	129.2, CH	7.20, m
	NH (4)		8.13, d(8.0)		8.15, d(8.5)
	29	169.1, C		169.0, C	
	30	22.4, CH_3_	1.69, s	22.4, CH_3_	1.69, s
	31			51.9, CH_3_	3.59, s

**Table 2:** ^1^H (500 MHz) and ^13^C (125 MHz) NMR data of compounds **3** in DMSO-*d*_6_ (*δ* in ppm).

Position	3	
	*δ*_C_, Type	*δ*_H_ (*J* in Hz)
1	177.2, C	
2	37.2, CH	2.49, m
3	44.2, CH_2_	1.97, dd(13.5, 8.0) 2.32, dd(13.5, 6.5)
4	132.3, C	
5	130.5, CH	5.57, s
6	130.2, C	
7	136.2, CH	4.96, d(9.5)
8	29.7, CH	2.49, m
9	40.3, CH_2_	1.29, m
10	35.7, CH	1.43, m
11	39.2, CH_2_	1.06, m
12	27.2, CH	1.51, m
13	44.4, CH_2_	0.83, m 1.19, m
14	31.0, CH	1.40, m
15	28.3, CH_2_	0.99, m 1.30, m
16	10.8, CH_3_	0.81, m
17	19.8, CH_3_	0.80, d(6.0)
18	20.9, CH_3_	0.80, d(6.0)
19	64.6, CH_2_	3.26, m
20	17.1, CH_3_	1.66, s
21	21.4, CH_3_	0.91, d(6.5)
22	17.2, CH_3_	1.69, s
23	16.4, CH_3_	0.99, d(7.0)

**Table 3:** Antimicrobial activity of activated compounds.

Compounds	Antibacterial (MIC, μg/mL)			Antifungal (MIC, μg/mL)		
	*S. aureus* ATCC 29213	MRSA GDMCC 1.1263	*A. baumannii* BNCC254392	*F. oxysporum* HNM 1003	*F. oxysporum* f. sp. *vasinfectum*	*A. longipes* BNCC119020
**3**	1	16	-	-	-	-
**4**	8	4	-	16	64	-
**5**	32	-	-	32	-	-
**6**	16	8	-	-	32	-
**10**	16	1	-	-	-	-
**12**	-	-	-	-	-	>128
**13**	0.5	0.5	32	16	4	64
**18**	128	-	-	-	-	-
Gentamicin	0.5	0.125	0.5	-	-	-
Nystatin	-	-	-	1	1	1

“-” means no MIC value tested.

## Data Availability

Data will be made available on request.

## Supplementary Materials

The following supporting information can be downloaded at: https://www.mdpi.com/article/10.3390/molecules31214034/s1, Figures S1–S19: ^1^H NMR, ^13^C NMR, HSQC, HMBC, COSY, HRESIMS and experimental ECD spectra of compounds **1**, **2** and **3**; Figures S20–S22: ITS1-5.8S-ITS2 sequences, the phylogenetic tree, and photo of *Penicillium* sp. SCSIO41052; Figure S23: Preliminary screening of antifungal activity of all compounds; Table S1: Marfey’s analyses of amino acid components in (**1**); Table S2: Preliminary screening of antibacterial activity of all compounds; Table S3: Acetylcholinesterase inhibition activity of compounds **3**–**6**; Table S4: Antitumour activity of compounds **1**–**2**, **8**–**12**.
