# Supplementary material for: Secondary Metabolites from Mariana Trench-Derived Penicillium sp. SCSIO41052 and Their Antimicrobial Activity
Source: Molecules. 2026 Nov 9;31(21):4034. doi: 10.3390/molecules31214034 (PMC13765202; doi:10.3390/molecules31214034)

Supplementary Data

Secondary Metabolites from Hadal Trench-Derived *Penicillium* sp. SCSIO41052 and their Antimicrobial Activity

Qinlin Cao[a,c], Xiaoyan Pang[a*], Yufan Zhang[a], Xin Su[a], Xueni Wang[b*], Junfeng Wang[a], Xuefeng Zhou[a], Bin Yang[a], Weijia Zhang[d], and Yonghong Liu[a,c*]

[a] *State Key Laboratory of Tropical Oceanography/Guangdong Key Laboratory of Marine Materia Medica, South China Sea Institute of Oceanology, Chinese Academy of Sciences, Guangzhou 510301, China*

[b]*Guangxi Engineering Research Center for High-Value Utilization of Guangxi-Produced Authentic medicinal Herbs, Institute of Traditional Chinese and Zhuang-Yao Ethnic Medicine, Guangxi University of Chinese Medicine, Nanning 530200, China*

[c] *University of Chinese Academy of Sciences, Beijing 100049, China*

[d] *Institute of Deep-sea Science and Engineering, Chinese Academy of Sciences, Sanya 572000, China*

* Correspondence:

xypang@scsio.ac.cn, Tel : +86-020-8902-3174 (X.P.);

wangxueni@gxtcmu.edu.cn, (X.W.)

yonghongliu@scsio.ac.cn, Tel: +86-020-8902-3244 (Y.L.).

Table of Contents

Fig. S1 ^{1}H NMR spectrum of **1** in DMSO-d_6 at 500 MHz. 1
Fig. S2 ^{13}C NMR spectrum of **1** in DMSO-d_6 at 125 MHz. 1
Fig. S3 HSQC spectrum of **1** in DMSO- d_6. 2
Fig. S4 HMBC spectrum of **1** in DMSO- d_6. 2
Fig. S5 HRESIMS spectrum of **1**. 3
Fig. S6 Experimental ECD spectra of **1**. 3
Fig. S7 ^{1}H NMR spectrum of **2** in DMSO-d_6 at 500 MHz. 4
Fig. S8 ^{13}C NMR spectrum of **2** in DMSO-d_6 at 125 MHz. 4
Fig. S9 ^{1}H-^{1}H COSY spectrum of **2** in DMSO- d_6. 5
Fig. S10 HSQC spectrum of **2** in DMSO- d_6. 5
Fig. S11 HMBC spectrum of **2** in DMSO- d_6. 6
Fig. S12 HRESIMS spectrum of **2**. 6
Fig. S13 Experimental ECD spectra of **2**. 7
Fig. S14 ^{1}H NMR spectrum of **3** in DMSO-d_6 at 500 MHz. 7
Fig. S15 ^{13}C NMR spectrum of **3** in DMSO-d_6 at 125 MHz. 8
Fig. S16 ^{1}H-^{1}H COSY spectrum of **3** in DMSO- d_6. 8
Fig. S17 HSQC spectrum of **3** in DMSO- d_6. 9
Fig. S18 HMBC spectrum of **3** in DMSO- d_6. 9
Fig. S19 HRESIMS spectrum of **3**. 10
Fig. S20 ITS1-5.8S-ITS2 sequences of *Penicillium* sp. SCSIO41052 10
Fig. S21 The phylogenetic tree of *Penicillium* sp. SCSIO41052 11
Fig. S22 The photo of *Penicillium* sp. SCSIO41052 11
Table S1. Marfey's analyses of amino acid components in (**1**) 12
Table S2. Preliminary screening of antibacterial activity of all compounds 14
Fig. S23 Preliminary screening of antifungal activity of all compounds 15
Table S3. Acetylcholinesterase inhibition activity of compounds **3**–**6** 15
Table S4. Antitumour activity of compounds **1**–**2**, **8**–**12** 16

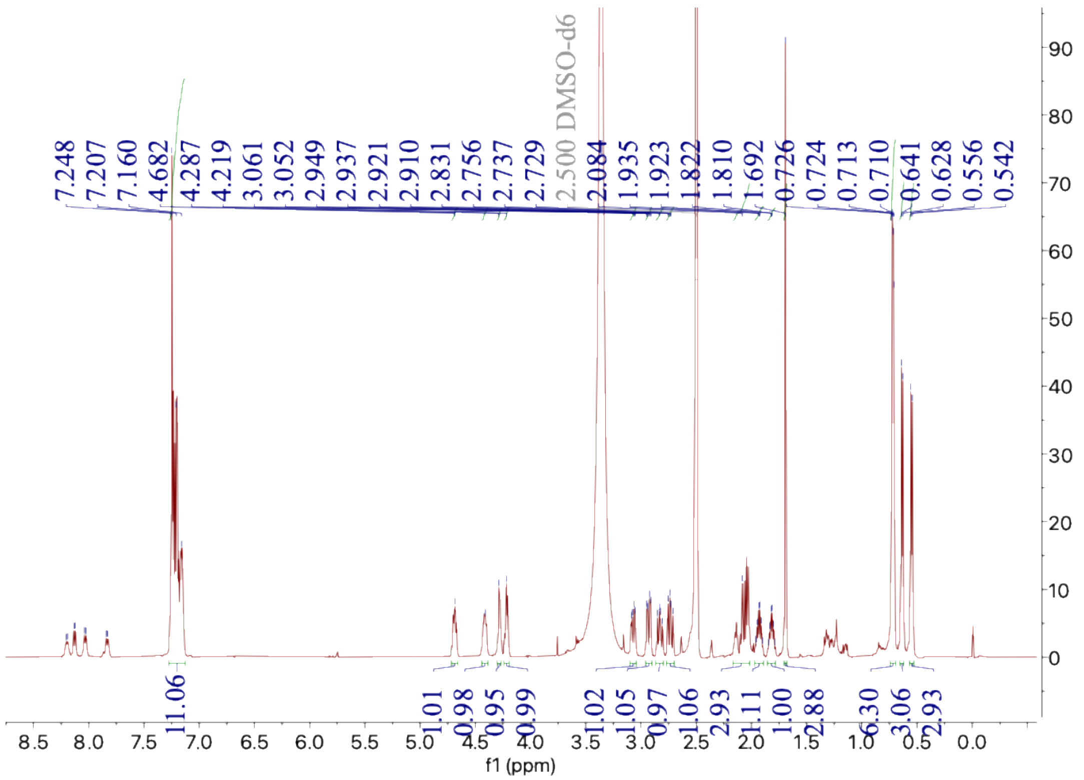

Figure S1 ^{1}H NMR spectrum of **1** in DMSO-d_6 at 500 MHz.

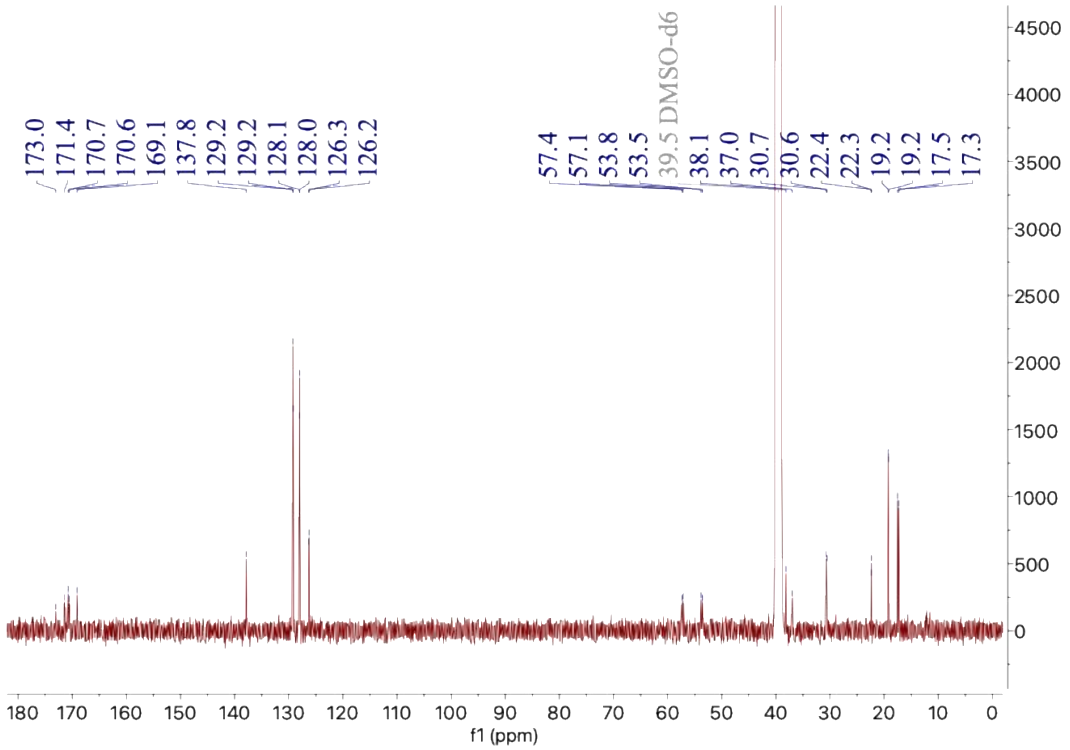

Figure S2 ^{13}C NMR spectrum of **1** in DMSO-d_6 at 125 MHz.

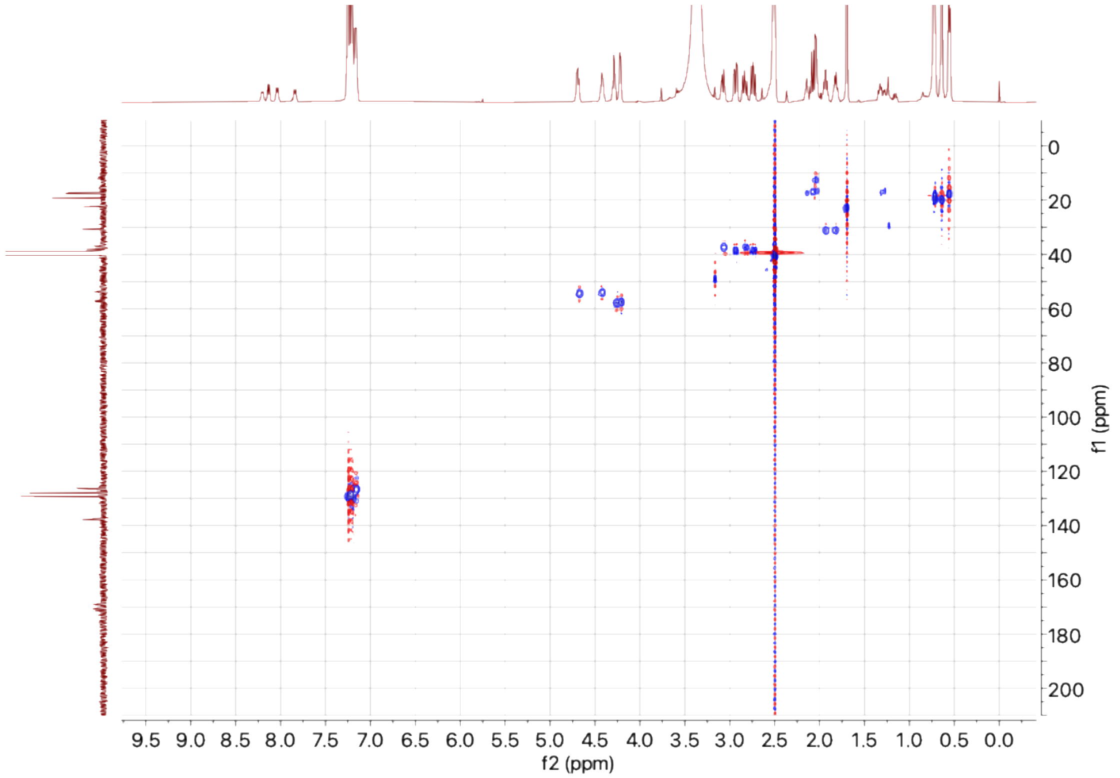

Figure S3 HSQC spectrum of **1** in DMSO- d_6.

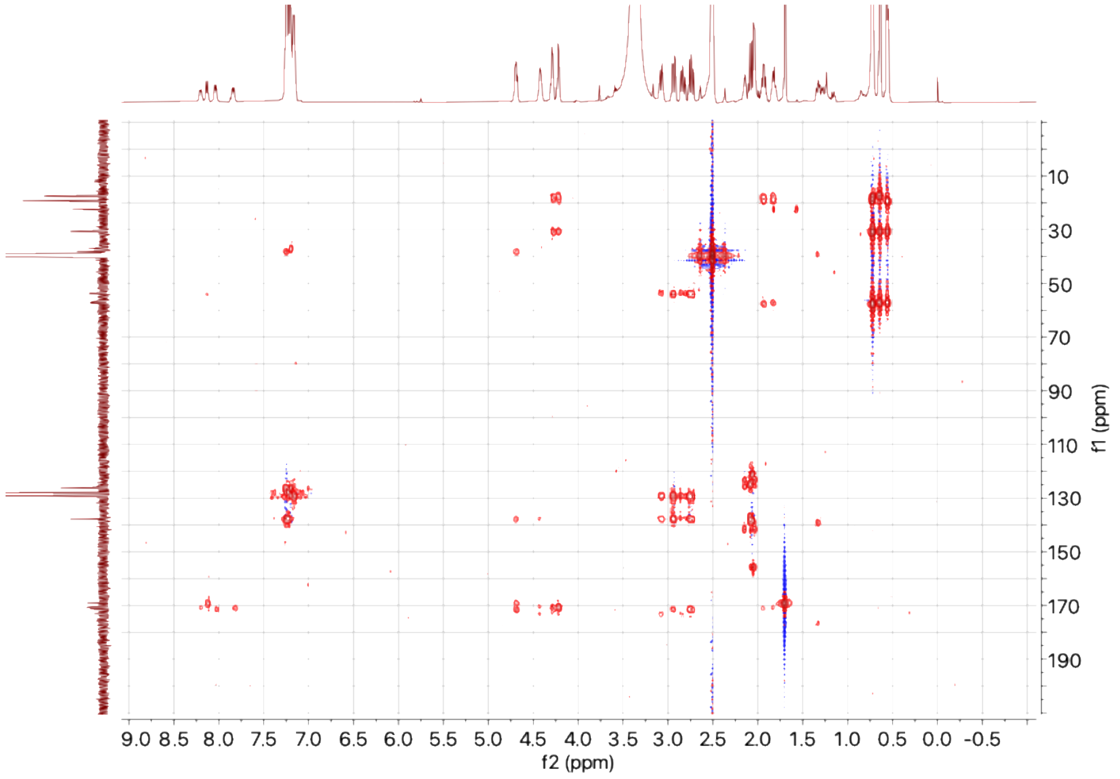

Figure S4 HMBC spectrum of **1** in DMSO- d_6.

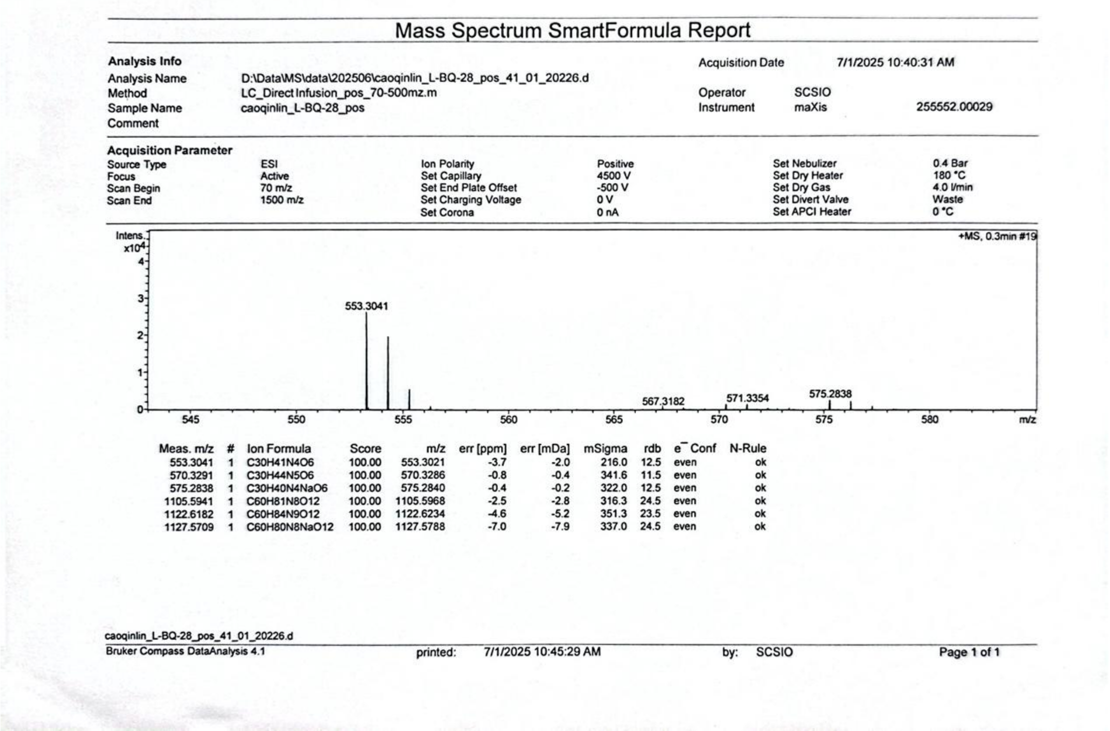

Mass Spectrum SmartFormula Report

Analysis Info

Analysis Name D:\Data\MS\data\202506\caoqinlin_L-BQ-28_pos_41_01_20226.d
Method LC_Direct Infusion_pos_70-500mz.m
Sample Name caoqinlin_L-BQ-28_pos
Comment

Acquisition Date 7/1/2025 10:40:31 AM
Operator SCSIO
Instrument maXis 255552.00029

Acquisition Parameter

Source Type	ESI	Ion Polarity	Positive	Set Nebulizer	0.4 Bar
Focus	Active	Set Capillary	4500 V	Set Dry Heater	180 °C
Scan Begin	70 m/z	Set End Plate Offset	-500 V	Set Dry Gas	4.0 l/min
Scan End	1500 m/z	Set Charging Voltage	0 V	Set Divert Valve	Waste
		Set Corona	0 nA	Set APCI Heater	0 °C

Meas. m/z	#	Ion Formula	Score	m/z	err [ppm]	err [mDa]	mSigma	rdb	e⁻ Conf	N-Rule
553.3041	1	C30H41N4O6	100.00	553.3021	-3.7	-2.0	216.0	12.5	even	ok
570.3291	1	C30H44N5O6	100.00	570.3286	-0.8	-0.4	341.6	11.5	even	ok
575.2838	1	C30H40N4NaO6	100.00	575.2840	-0.4	-0.2	322.0	12.5	even	ok
1105.5941	1	C60H81N8O12	100.00	1105.5968	-2.5	-2.8	316.3	24.5	even	ok
1122.6182	1	C60H84N9O12	100.00	1122.6234	-4.6	-5.2	351.3	23.5	even	ok
1127.5709	1	C60H80N8NaO12	100.00	1127.5788	-7.0	-7.9	337.0	24.5	even	ok

caoqinlin_L-BQ-28_pos_41_01_20226.d
Bruker Compass DataAnalysis 4.1 printed: 7/1/2025 10:45:29 AM by: SCSIO Page 1 of 1

Figure S5 HRESIMS spectrum of **1**.

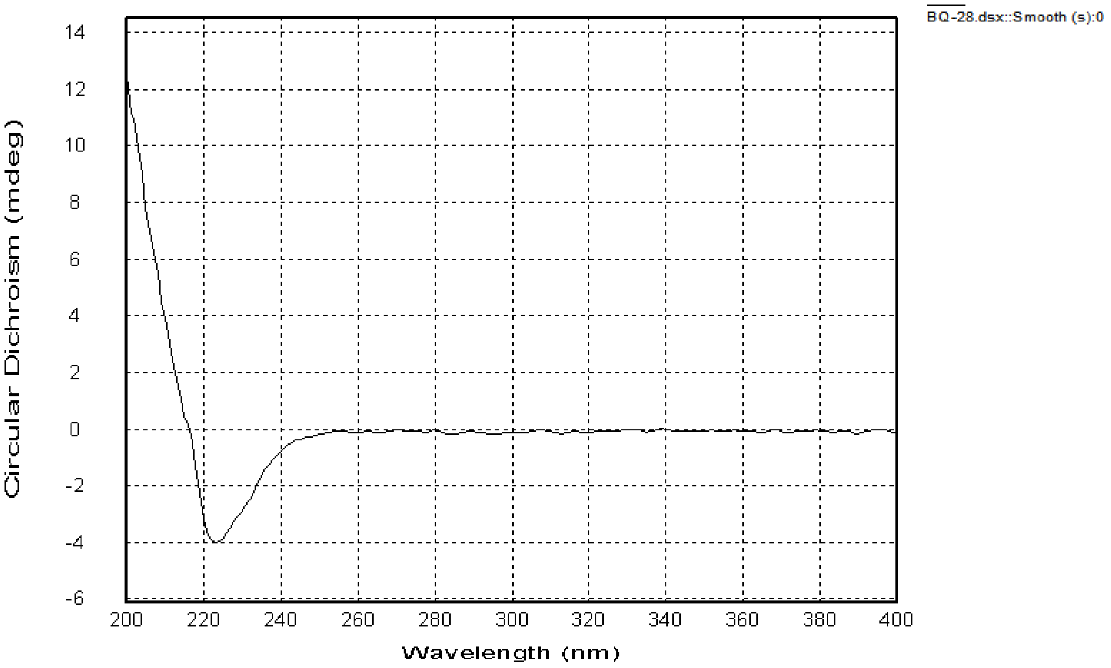

Figure S6 Experimental ECD spectra of **1**.

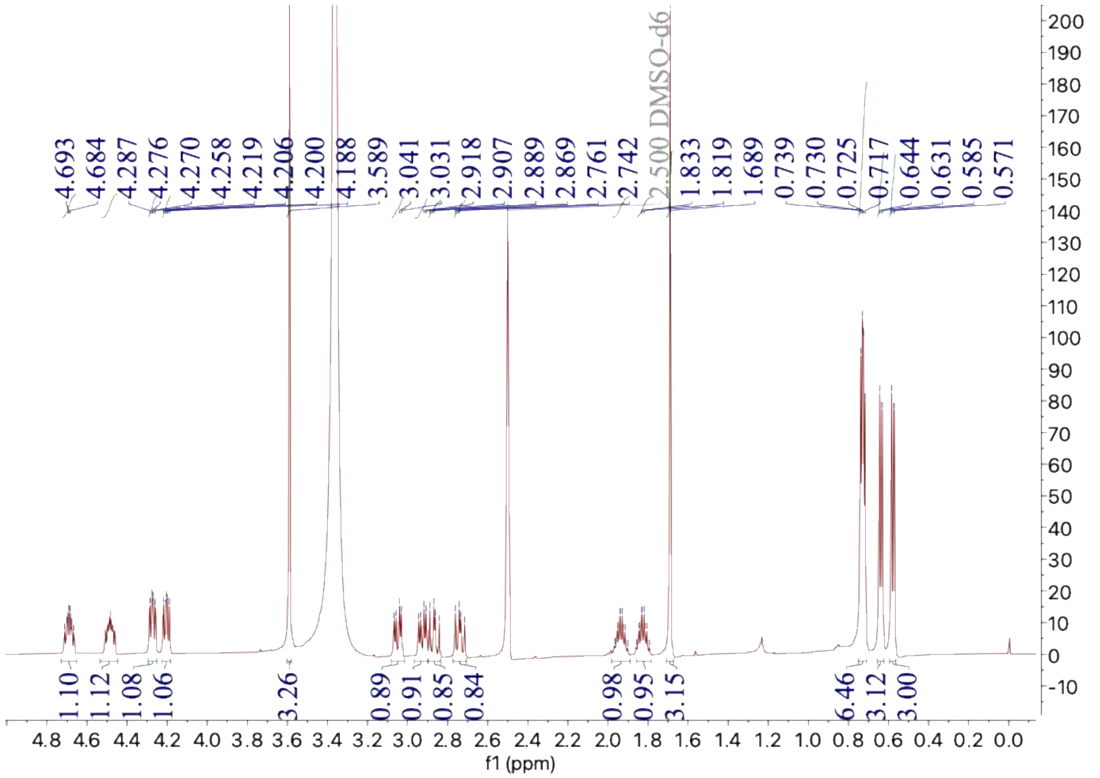

Figure S7 ^{1}H NMR spectrum of **2** in DMSO-d_6 at 500 MHz.

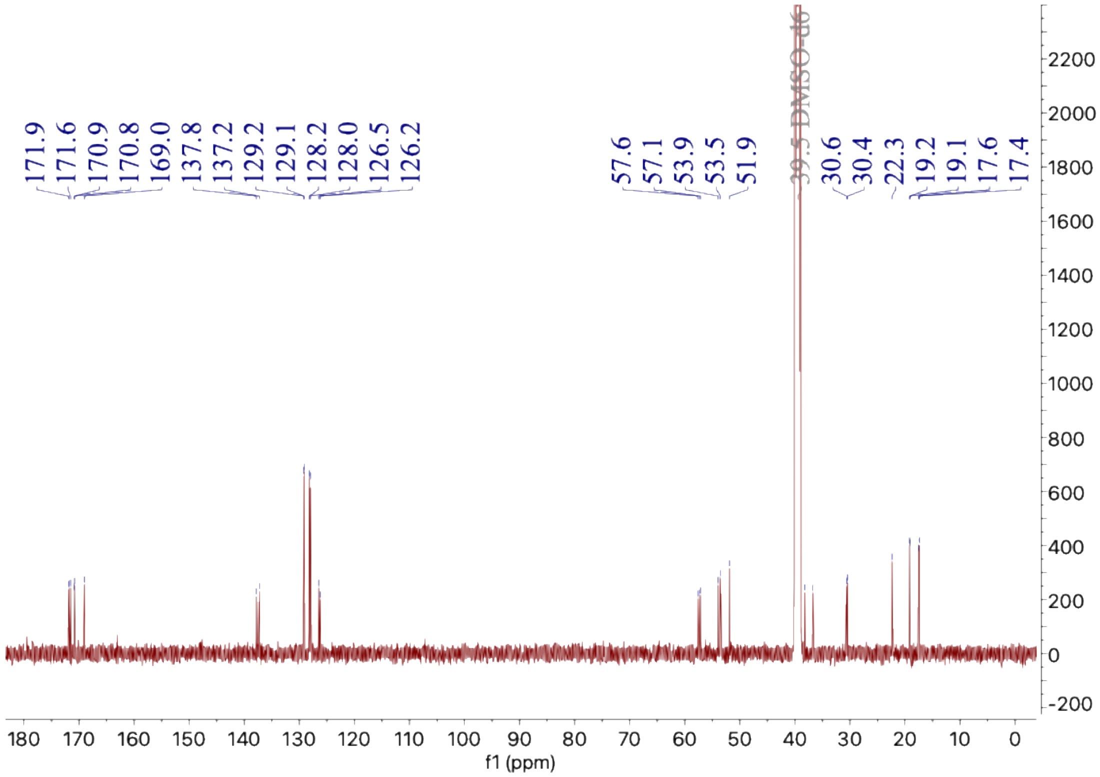

Figure S8 ^{13}C NMR spectrum of **2** in DMSO-d_6 at 125 MHz.

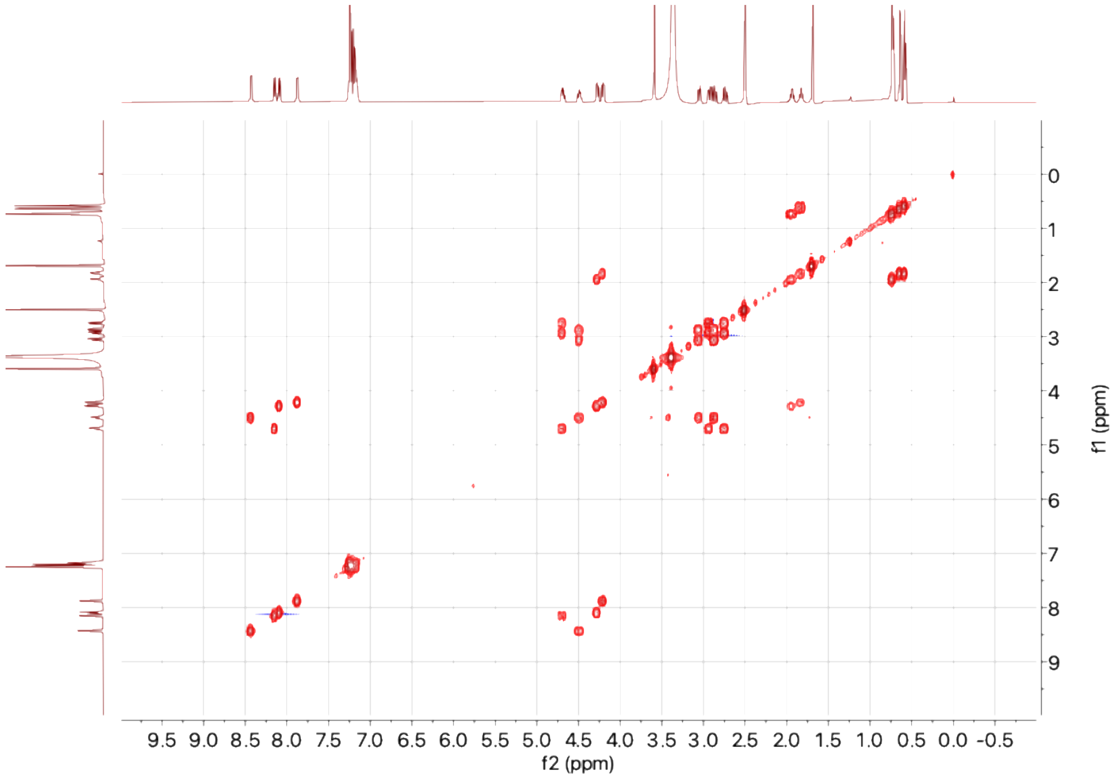

Figure S9 ^{1}H-^{1}H COSY spectrum of **2** in DMSO- d_6.

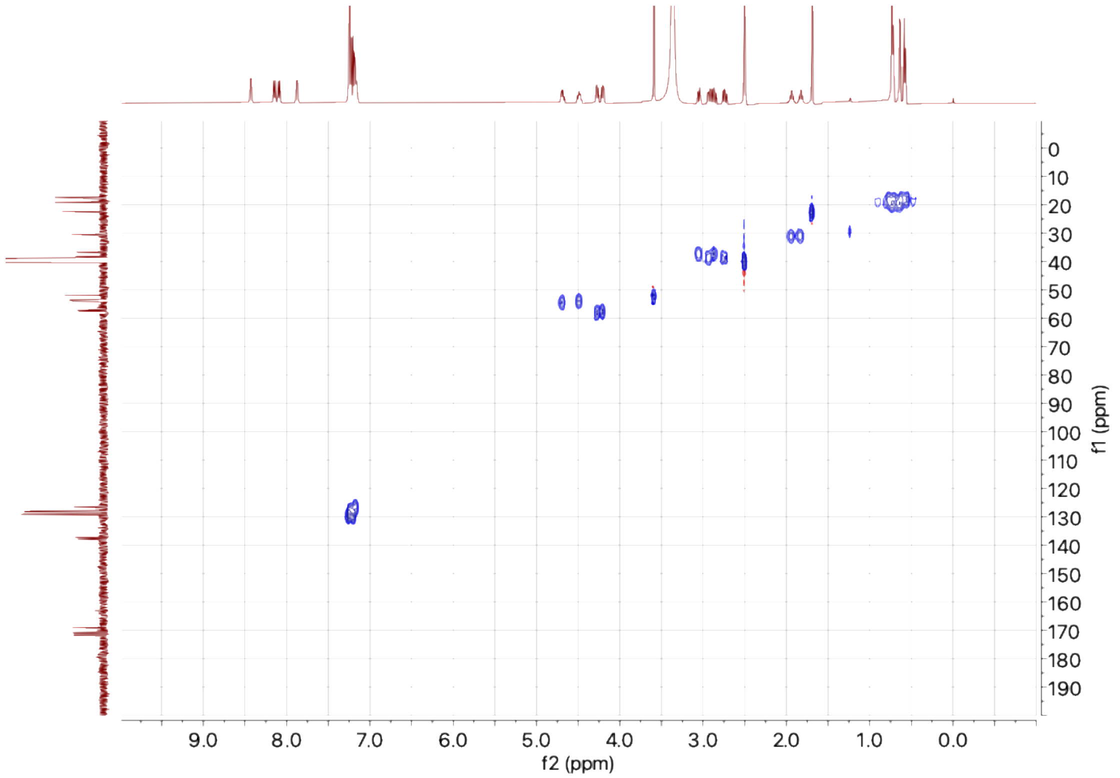

Figure S10 HSQC spectrum of **2** in DMSO- d_6.

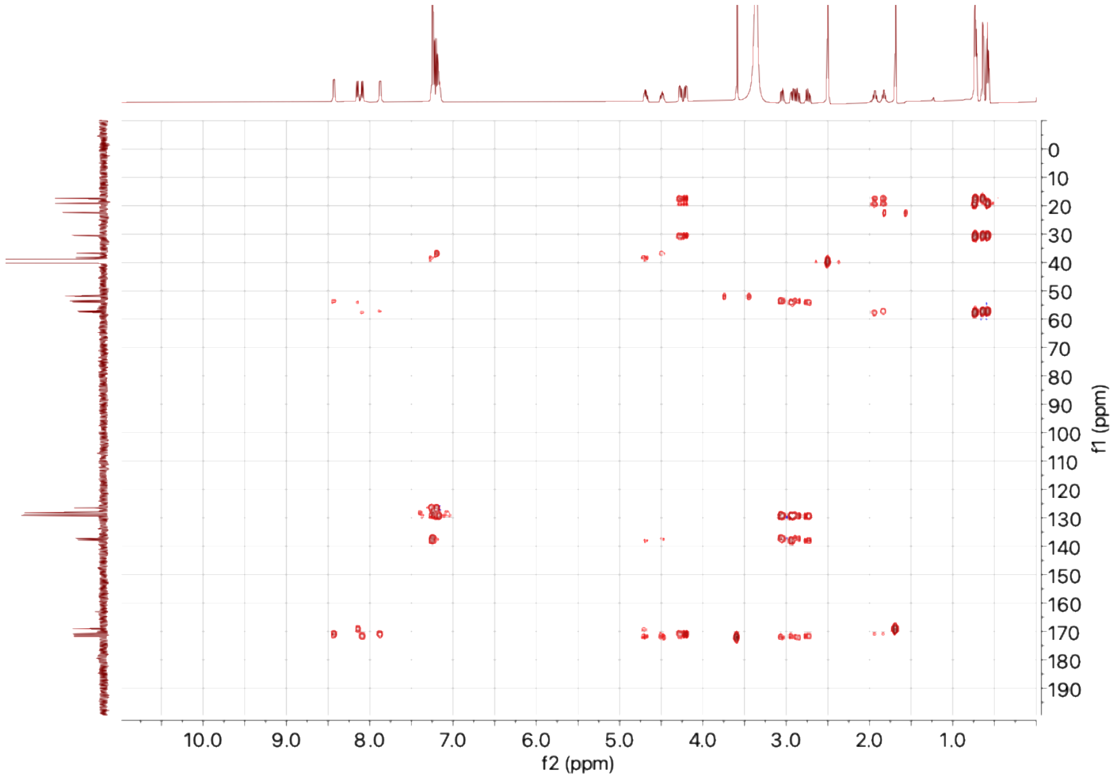

Figure S11 HMBC spectrum of **2** in DMSO- d_6.

Mass Spectrum SmartFormula Report

Analysis Info

Analysis Name	D:\Data\MS\data\202506\caoqinlin_L-BQ-29_pos_2_01_20037.d	Acquisition Date	6/5/2025 10:54:40 AM	
Method	LC_Direct Infusion_pos_70-500mz.m	Operator	SCSIO	
Sample Name	caoqinlin_L-BQ-29_pos	Instrument	maXis	255552.00029
Comment				

Acquisition Parameter

Source Type	ESI	Ion Polarity	Positive	Set Nebulizer	0.4 Bar
Focus	Active	Set Capillary	4500 V	Set Dry Heater	180 °C
Scan Begin	70 m/z	Set End Plate Offset	-500 V	Set Dry Gas	4.0 l/min
Scan End	1500 m/z	Set Charging Voltage	0 V	Set Divert Valve	Waste
		Set Corona	0 nA	Set APCI Heater	0 °C

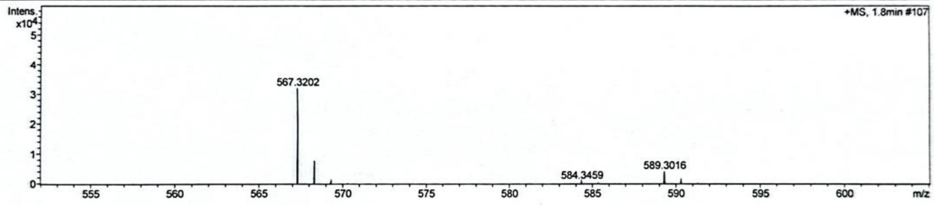

Meas. m/z	#	Ion Formula	Score	m/z	err [ppm]	err [mDa]	mSigma	rdb	e⁻ Conf	N-Rule
567.3202	1	C31H43N4O6	100.00	567.3177	-4.5	-2.5	61.1	12.5	even	ok
584.3459	1	C31H46N5O6	100.00	584.3443	2.8	1.7	12.9	11.5	even	ok
589.3016	1	C31H42N4NaO6	100.00	589.2997	-3.2	-1.9	33.3	12.5	even	ok

caoqinlin_L-BQ-29_pos_2_01_20037.d

Bruker Compass DataAnalysis 4.1 printed: 6/5/2025 11:01:28 AM by: SCSIO Page 1 of 1

Figure S12 HRESIMS spectrum of **2**.

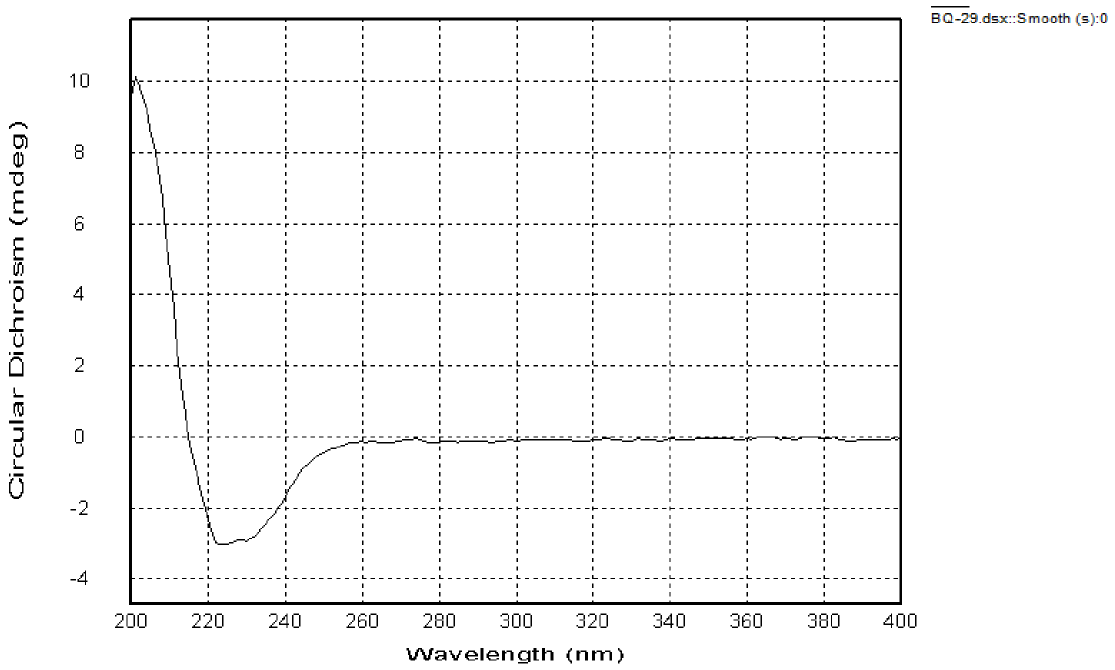

Figure S13 Experimental ECD spectra of **2**.

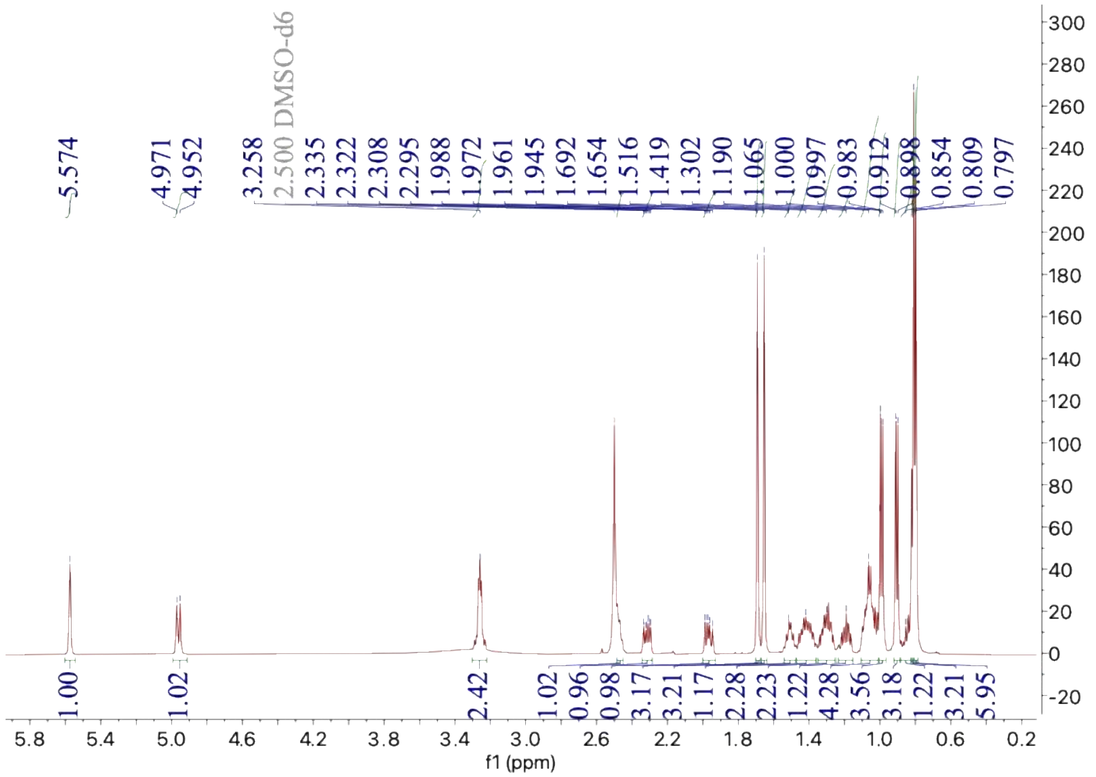

Figure S14 ^{1}H NMR spectrum of **3** in DMSO-d_6 at 500 MHz.

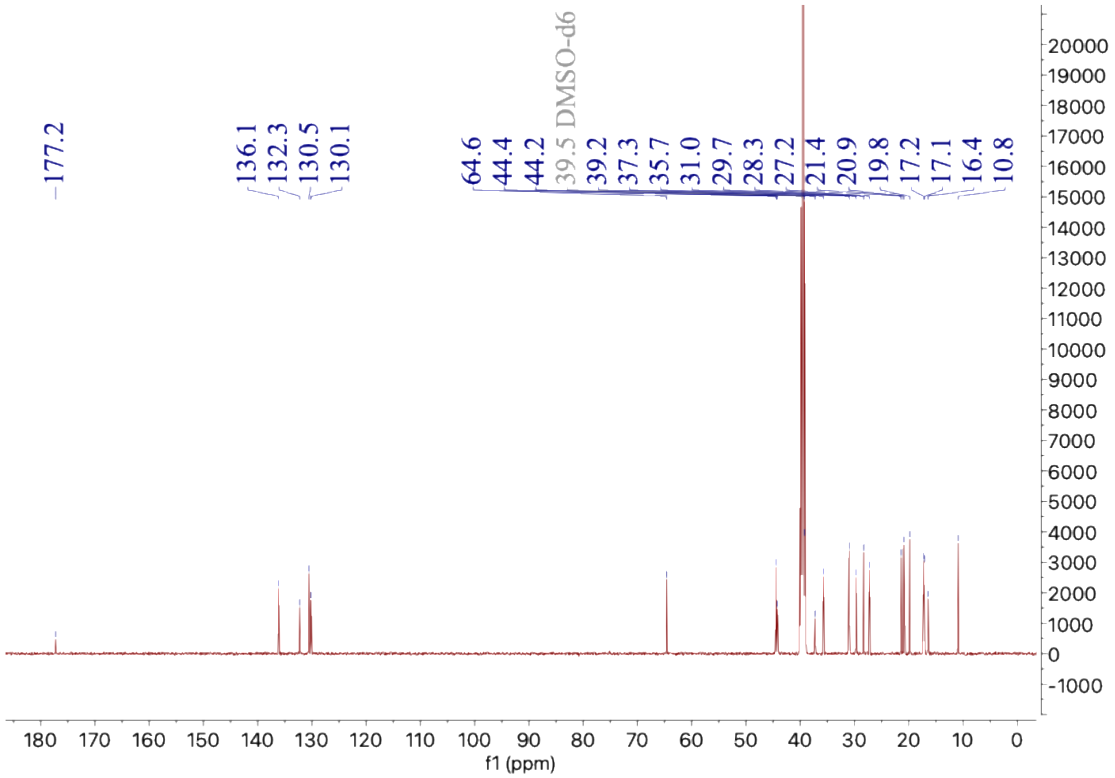

Figure S15 ^{13}C NMR spectrum of **3** in DMSO-d_6 at 125 MHz.

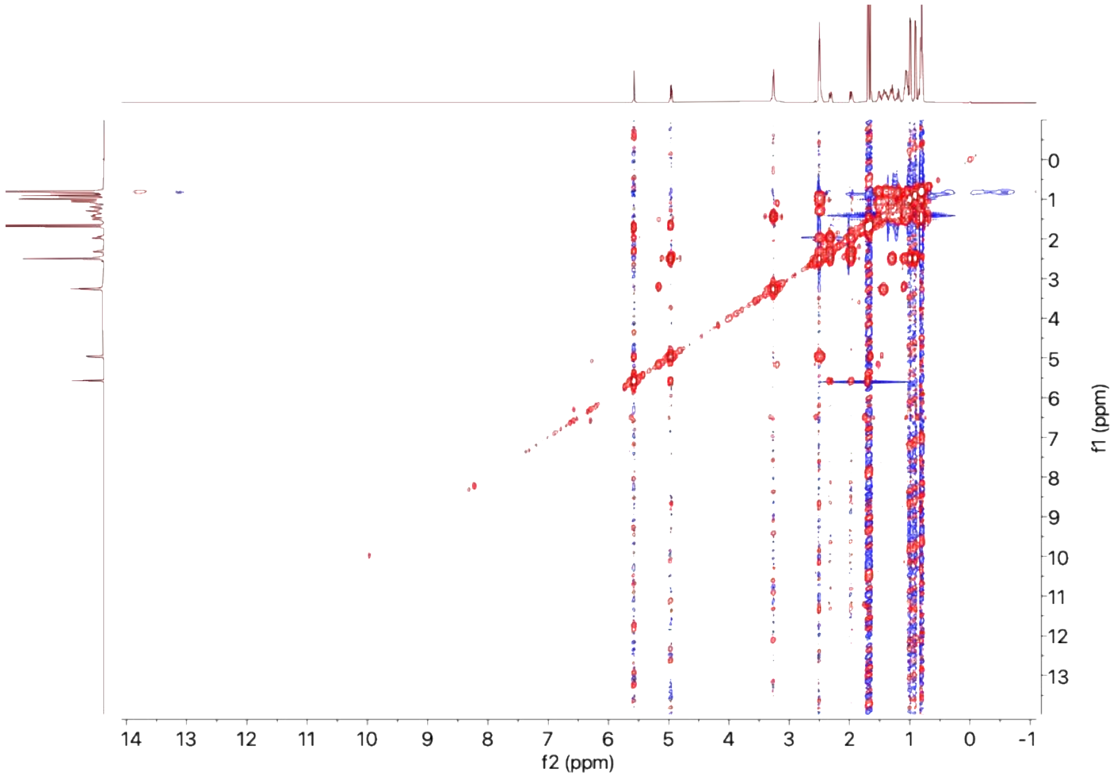

Figure S16 ^{1}H-^{1}H COSY spectrum of **3** in DMSO- d_6.

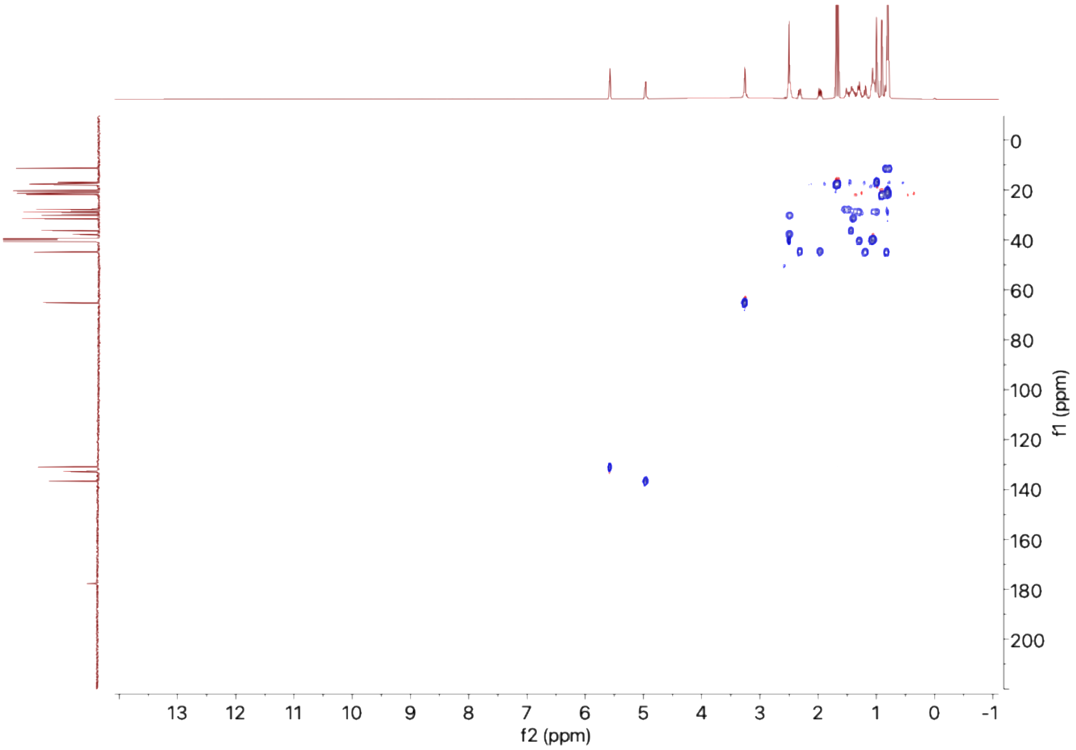

Figure S17 HSQC spectrum of **3** in DMSO- d_6.

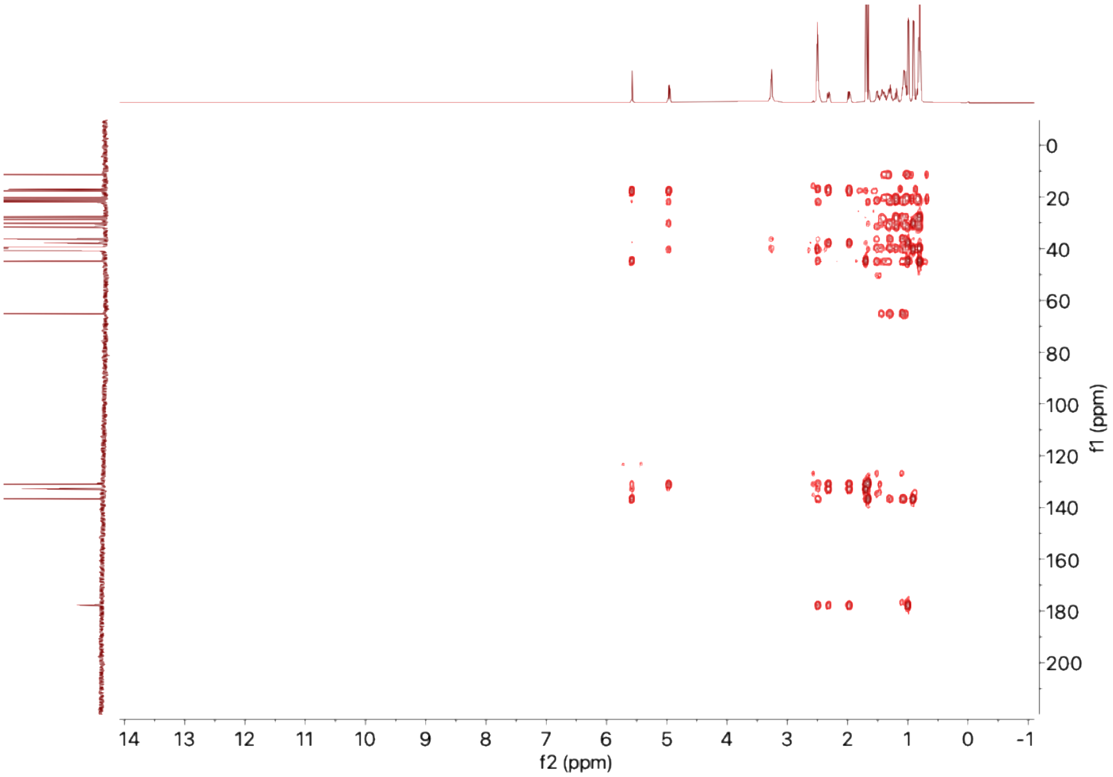

Figure S18 HMBC spectrum of **3** in DMSO- d_6.

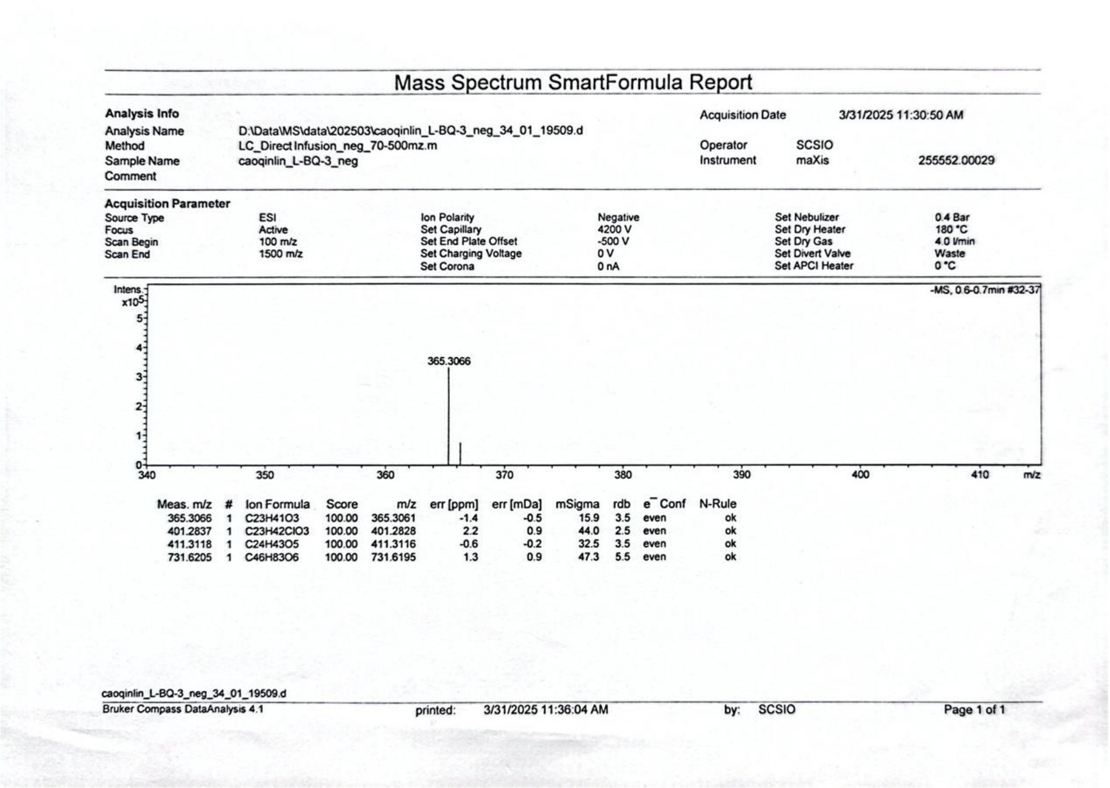

Figure S19 HRESIMS spectrum of **3**.

>SCSIO41052
TGCGGAAGGATCATTACCGAGTGAGGGCCCTCTGGGTCCAACCTCCCACCCGTGT
TTATCGTACCTTGTTGCTTCGGCGGGCCCGCCTCACGGCCGCCGGGGGGCATCCGC
CCCCGGGCCCGCGCCCGCCGAAGACACACAAACGAACTCTTGTCTGAAGATTGCA
GTCTGAGTACTTGACTAAATCAGTTAAAACTTTCAACAACGGATCTCTTGGTTCCG
GCATCGATGAAGAACGCAGCGAAATGCGATAAGTAATGTGAATTGCAGAATTCAGT
GAATCATCGAGTCTTTGAACGCACATTGCGCCCCCTGGTATTCCGGGGGGCATGCC
TGTCCGAGCGTCATTGCTGCCCTCAAGCACGGCTTGTGTGTTGGGCTCTCGCCCCC
CGCTTCCGGGGGGCGGGCCCGAAAGGCAGCGGCGGCACCGCGTCCGGTCCTCGA
GCGTATGGGGCTTCGTCACCCGCTCTGTAGGCCCGGCCGGCGCCCGCCGGCGAAC
ACCATCAATCTTAACCAGGTTGACCTCGGATCAGGTAGGGATACCCGCTGAACTTA
AGCATATC

Figure S20 *Penicillium* sp. SCSIO41052 ITS1-5.8S-ITS2 sequences

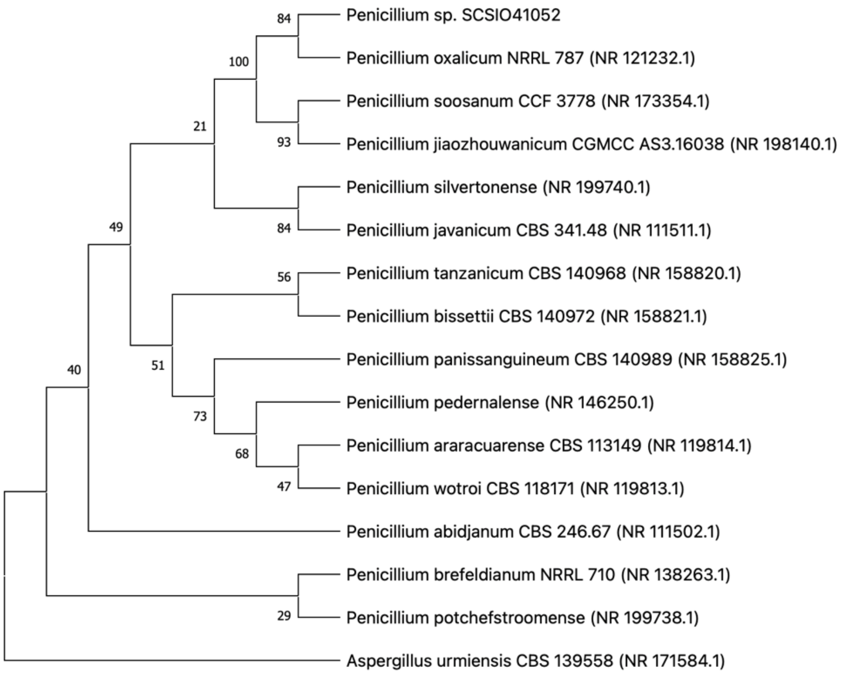

Figure S21 The phylogenetic tree of *Penicillium* sp. SCSIO41052

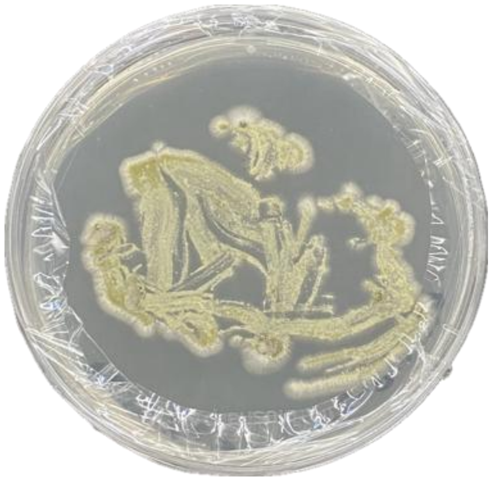

Figure S22 The photo of *Penicillium* sp. SCSIO41052

Table S1. Marfey's analyses of amino acid components in (**1**)

	Retention Time (min)			
Amino Acids	D standard	L standard	**1**	Absolute Configuration
Val	51.8	46.2	51.8	D
Phe	55.8	52.1	55.8	D

Conditions: column, Cosmosil 5C$_{18}$-MS-II column (φ10 × 250 mm); flow rate 2.0 mL/min; detection, UV 390 nm; solvent, mobile phase A (0.3% formic acid in water), mobile phase B (ACN); gradient: 0–60.0 min, 10–60% B; 60.1–67.0 min, 10% B.

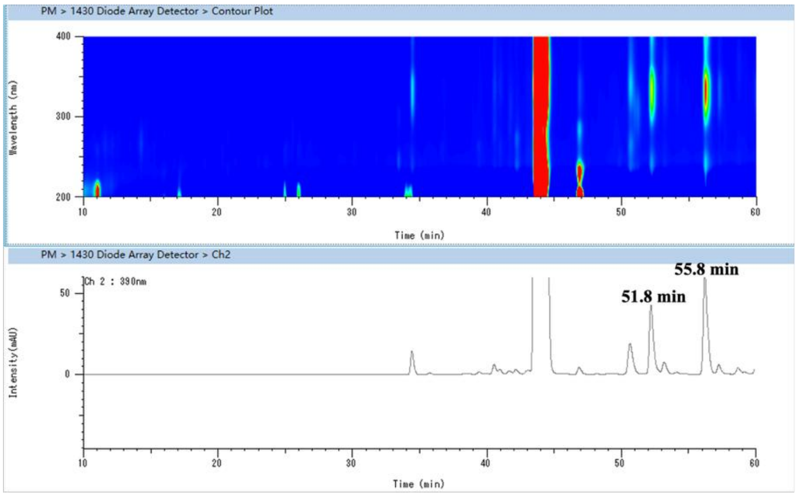

D-Val and D-Phe from **1**

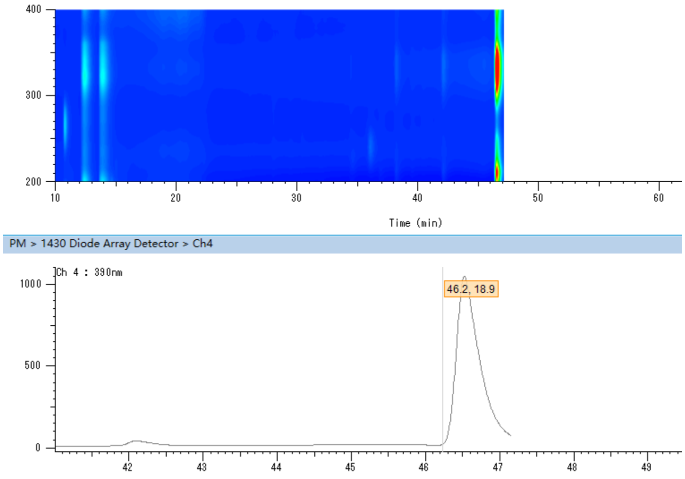

L-Val standard

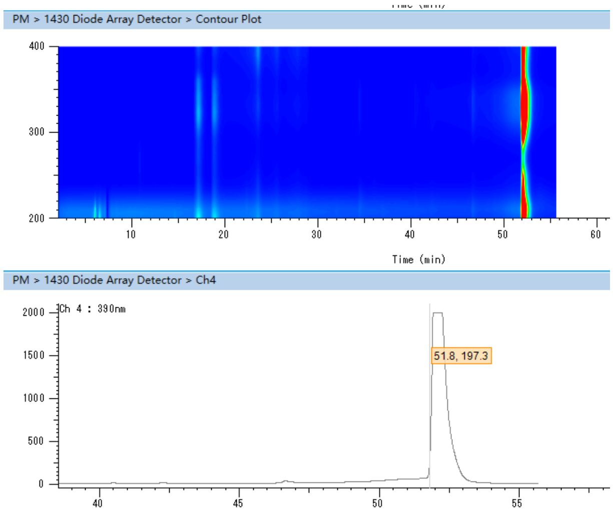

D-Val standard

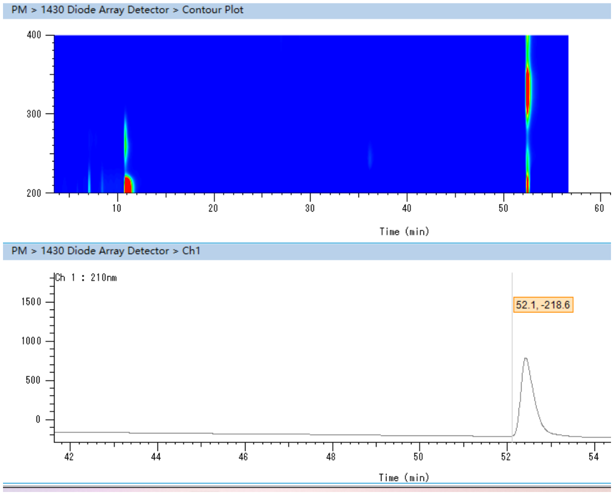

L-Phe standard

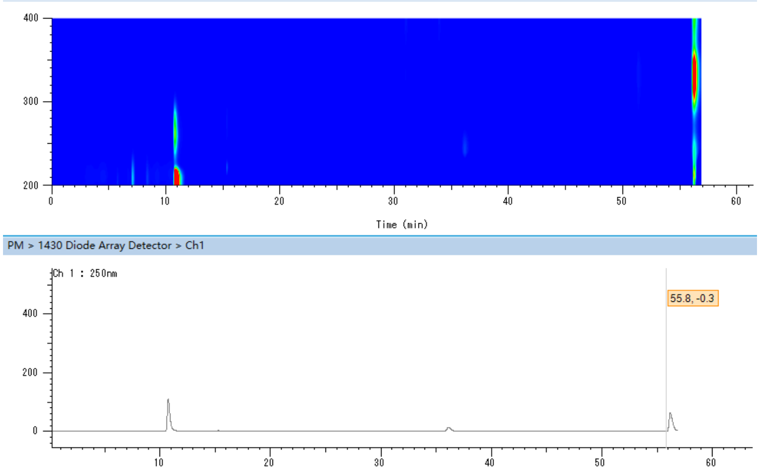

D-Phe standard

Table S2. Preliminary screening of antibacterial activity of all compounds

**Compounds**	**Antibacterial** (inhibition zone, **cm)**				
	S. aureus	**MRSA**	***A. baumannii***	***K. pneumoniae***	***E. faecalis***
**1**	no inhibition zone	no inhibition zone	no inhibition zone	no inhibition zone	no inhibition zone
**2**	no inhibition zone	no inhibition zone	no inhibition zone	no inhibition zone	no inhibition zone
**3**	1.2	0.8	no inhibition zone	no inhibition zone	no inhibition zone
**4**	1.2	1.0	no inhibition zone	no inhibition zone	no inhibition zone
**5**	0.9	no inhibition zone	no inhibition zone	no inhibition zone	no inhibition zone
**6**	0.9	0.8	no inhibition zone	no inhibition zone	no inhibition zone
**7**	no inhibition zone	no inhibition zone	no inhibition zone	no inhibition zone	no inhibition zone
**8**	no inhibition zone	no inhibition zone	no inhibition zone	no inhibition zone	no inhibition zone
**9**	no inhibition zone	no inhibition zone	no inhibition zone	no inhibition zone	no inhibition zone

**10**	1.1	1.1	no inhibition zone	no inhibition zone	no inhibition zone
**11**	no inhibition zone	no inhibition zone	no inhibition zone	no inhibition zone	no inhibition zone
**12**	no inhibition zone	no inhibition zone	no inhibition zone	no inhibition zone	no inhibition zone
**13**	1.4	1.8	0.7	no inhibition zone	no inhibition zone
**14**	no inhibition zone	no inhibition zone	no inhibition zone	no inhibition zone	no inhibition zone
**15**	no inhibition zone	no inhibition zone	no inhibition zone	no inhibition zone	no inhibition zone
**16**	no inhibition zone	no inhibition zone	no inhibition zone	no inhibition zone	no inhibition zone
**17**	no inhibition zone	no inhibition zone	no inhibition zone	no inhibition zone	no inhibition zone
**18**	0.8	no inhibition zone	no inhibition zone	no inhibition zone	no inhibition zone
Gentamicin	1.5	1.3	1.8	2.0	1.2

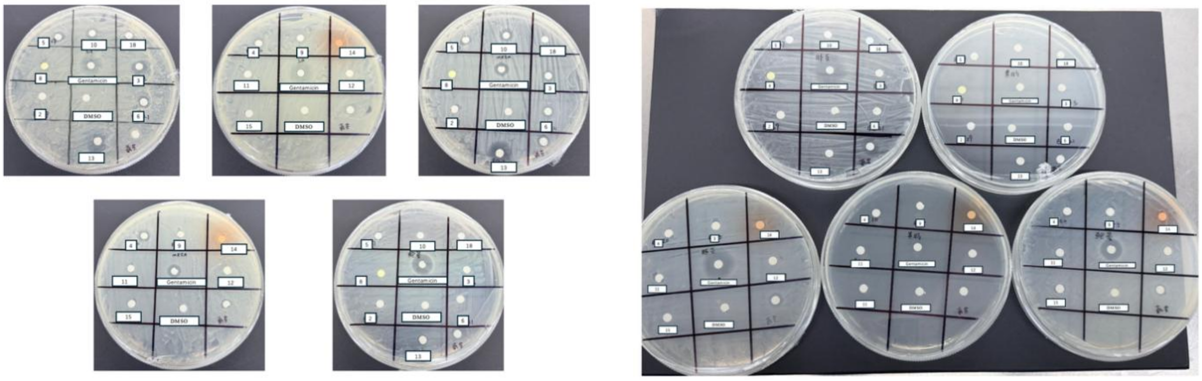

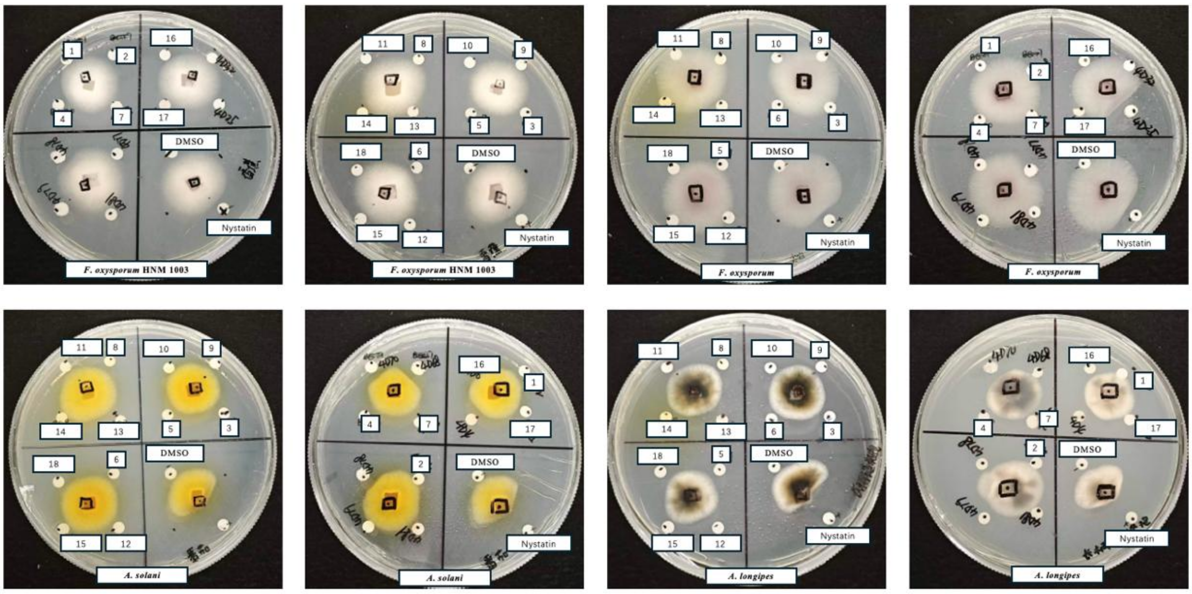

Figure S23 Preliminary screening of antifungal activity of all compounds

Table S3. Acetylcholinesterase inhibition activity of compounds **3–6**

Compounds (50 μg/ml)	Inhibition rate (%)
**3**	52.1
**4**	38.1
**5**	61.5
**6**	58.2
Tacrine	76.4

Table S4. Antitumour activity of compounds **1–2, 8–12**

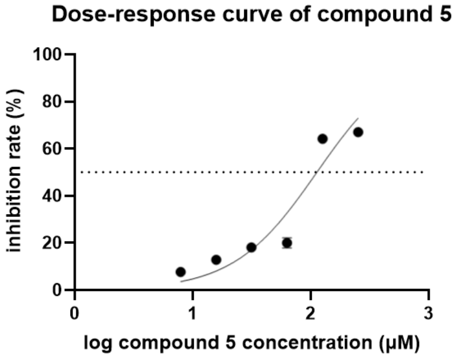

SD: 26.65; SEM: 10.88

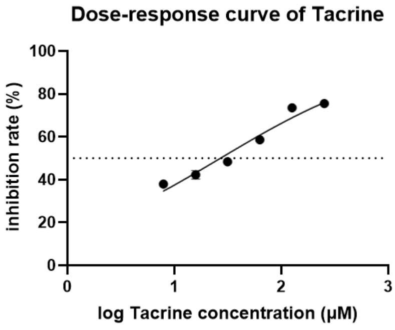

SD: 15.95; SEM: 6.512

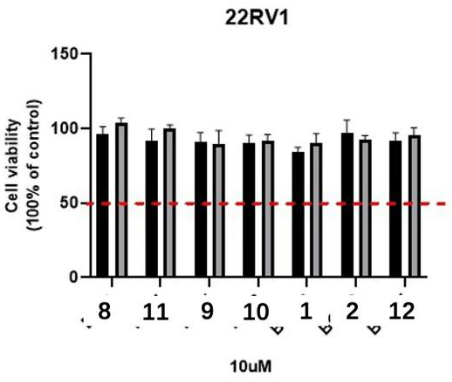

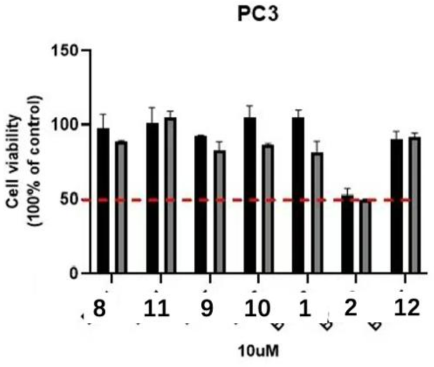

Supplement: Supplementary file 1 [file molecules-31-04034-s001.zip › molecules-4610466-supplementary.pdf]
